# Preliminary Identification and Quantification of Individual Polyphenols in *Fallopia japonica* Plants and Honey and Their Influence on Antimicrobial and Antibiofilm Activities

**DOI:** 10.3390/plants13131883

**Published:** 2024-07-08

**Authors:** Alexandra-Antonia Cucu, Adriana Cristina Urcan, Otilia Bobiș, Victorița Bonta, Mihaiela Cornea-Cipcigan, Adela Ramona Moise, Ștefan Dezsi, Claudia Pașca, Gabriela-Maria Baci, Daniel Severus Dezmirean

**Affiliations:** 1 Faculty of Animal Science and Biotechnology, University of Animal Sciences and Veterinary Medicine Cluj Napoca, 400372 Cluj-Napoca, Romania; antonia.cucu@usamvcluj.ro (A.-A.C.); obobis@usamvcluj.ro (O.B.); adela.moise@usamvcluj.ro (A.R.M.); claudia.pasca@usamvcluj.ro (C.P.); gabriela-maria.baci@usamvcluj.ro (G.-M.B.); ddezmirean@usamvcluj.ro (D.S.D.); 2 Department of Microbiology and Immunology, Faculty of Animal Science and Biotechnologies, University of Agricultural Sciences and Veterinary Medicine, 400372 Cluj-Napoca, Romania; 3 Department of Horticulture and Landscaping, Faculty of Horticulture, University of Agricultural Sciences and Veterinary Medicine, 400372 Cluj-Napoca, Romania; mihaiela.cornea@usamvcluj.ro; 4 Faculty of Geography, Babeş-Bolyai University, 400084 Cluj-Napoca, Romania; stefan.dezsi@ubbcluj.ro

**Keywords:** *Fallopia japonica* honey, *Reynoutria japonica*, polyphenols, resveratrol, antimicrobial potential, antibiofilm activity

## Abstract

*Fallopia japonica* (FJ), an invasive plant species known for its rich bioactive compounds, has been used for centuries in traditional Chinese medicine. Despite its significant beekeeping potential, this aspect of FJ remains underexplored. This research aims to investigate the antimicrobial and antibiofilm properties of FJ plants and honey. Notably, this study is the first to identify individual phenolic compounds in both FJ plant tissues and FJ honey, highlighting resveratrol as a marker of FJ honey. The study tested inhibitory activity against seven bacterial strains: *Staphylococcus aureus*, *Enterococcus faecalis*, *Escherichia coli*, *Pseudomonas aeruginosa*, *Bacillus cereus*, *Salmonella enteritidis*, and the yeast *Candida albicans*. Disk diffusion and microdilution methods were used to assess antimicrobial activity, while the crystal violet staining test evaluated antibiofilm activity. Results showed that FJ plant tissues and honey exhibited strong inhibition, particularly against Gram-negative bacterial strains. The most significant inhibition of biofilm formation, by both FJ plant tissues and honey, was observed against *Staphylococcus aureus* and *Escherichia coli*. A significant positive correlation was found between antimicrobial activity and individual polyphenols, especially resveratrol. The antibacterial and antibiofilm potential of FJ plant tissues and honey suggests promising applications in sustainable beekeeping. Further research is necessary to evaluate the bioactive compounds found in FJ honey and their health effects.

## 1. Introduction

In recent years, there has been a notable worldwide shift towards the utilization of natural products that, once consumed, could promote balance and enhance health [1].

This trend stems from consumer concerns about the safety of synthetic compounds in processed food items and the negative impact these may induce on human health, compared with products that have natural sources—such as plants or bee products—and that are cost effective, rich in nutrients, full of biologically active compounds, and highly bioavailable for human consumption [2,3,4,5,6]. Thus, consumers are more interested in the so-called “functional foods” containing “ingredients that provide health benefits beyond the food’s basic nutritional components” [7] (p. 2).

Since ancient times, plants and honey have maintained a significant presence in human life due to their nutritional and health advantages [8,9,10]. Despite advancements in the pharmaceutical sector, these two abundant natural sources continue to hold significance as alternative medicine options.

Given this, *Fallopia japonica* (FJ) (*Polygonum cuspidatum*, *Reynoutria japonica*), commonly known as Japanese knotweed, has been employed in the Chinese traditional medicine for hundreds of years [11], for treating disorders such as cough, asthma, atherosclerosis, dermatitis, hypertension, gonorrhea, or cancer [12,13]. A plant originating from East Asia, it was introduced to Europe in the 1800s for horticultural purposes [14], becoming, nowadays, one of the most invasive species worldwide [15,16,17,18]. It is characterized as a perennial plant that forms dense tickets reaching heights of up to 3 m [19]. Its invasiveness its strongly influenced by its capacity to rapidly thrive in diverse habitats (pastures, riparian habitats, disturbed lands) [20,21] because of some unique characteristics, namely, both clonal and sexual reproduction capabilities [22], the potential for hybridization [23], significant environmental resilience [24,25], and an inhibitive mechanism towards native vegetation through its chemical composition [26]. Therefore, its resilience against eradication is very challenging and expensive to deal with [27,28,29,30]. Consequently, it poses a serious threat to the native flora and ecosystems it invades, and the FJ plant is increasingly being seen as a threat to global sustainability [31].

While its negative impact is acknowledged [31,32,33], the beneficial applications [34,35,36] and health traits [37,38,39] of this *Polygonaceae* species have captured the attention of the research community.

In this light, different anatomic parts of FJ plants have been extensively studied for their phytochemical composition, reveling important amounts of biological active compounds with therapeutic effects on human health, such as anthraquinones (emodin, citreorosein, physcion, etc.), flavonoids (rutin, quercetin, apigenin, kaempferol, isoquercitrin, etc.), stilbenes (resveratrol and polydatin), and various other compounds [40,41,42,43,44,45,46,47]. Yet, the most researched compounds, linked to the majority of the pharmacological effects, have been resveratrol, emodin, and polydatin [37,48,49,50,51,52,53,54,55,56]. Recent findings indicate that polyphenols, notably resveratrol, primarily found in grapes, red berries, or nuts, possess the capacity to stimulate cellular differentiation, resulting in the emergence of diverse antioxidant, antitumor, and neurogenic characteristics [57,58,59].

The antimicrobial efficacy of the FJ plant has been extensively studied, since FJ is considered one of the most important plants in Chinese herbal medicine, acknowledged as a natural resource of resveratrol [19]. This compound exhibits a wide spectrum of health-enhancing properties [6,60] and is regarded as mainly responsible for the pharmacological effects of the FJ plant [43,47,60,61,62,63].

For the need to find potential natural preservatives or new agents with high potential for disease prophylaxis, researchers have observed the antimicrobial and antibiofilm effects of many phenolic compounds derived from FJ plants [54,64,65,66,67,68,69].

Treating biofilm-associated infections poses a formidable challenge for clinicians, due to their resilience against most conventional antifungal medications. In this light, phytochemicals offer a promising alternative to antibiotics for addressing infections, due to their lower risk of inducing bacterial resistance [70]. Plants produce a diverse array of compounds (secondary metabolites) as part of their defense mechanisms against both biotic and abiotic stresses, the most important being the so-called phytoalexins (including the flavonoids, polyphenols, terpenoids, and glycosides) [71].

On the other hand, honey has throughout history been recognized for its antimicrobial properties [72], primarily attributed to its sugar composition, moisture level, osmotic pressure, pH, acidity, protein content, methylglyoxal, and bee peptides (the peptide defensin-1), all of which act together to inhibit bacterial growth [73,74]. Furthermore, some other compounds such as phenolic acids, and especially polyphenols and flavonoids, contribute significantly to this characteristic [75,76,77,78,79]. According to the literature, phenolic compounds demonstrate a clearly defined antibacterial mechanism by effectively reducing antibiotic resistance in infectious bacteria and hindering biofilm formation [80]. As a consequence, honey is widely regarded as one of the most promising treatments for infections associated with biofilms, due to its ability to inhibit both the formation of biofilms and the growth of pre-existing ones [81,82]. Moreover, taking in consideration the fact that the geographical origin, floral source, and phytochemical profile of the raw material is frequently linked to the bioactivity of honey [83,84], the FJ plant represents a species that, to date, has been under-appreciated regarding its beekeeping potential. Based on the most accurate understanding currently available, there have been no studies that have reported the presence of bioactive constituents in *Fallopia japonica* honey (FJH). FJH is a unique type of honey, as yet underexplored. There have been only a few studies, all of them carried out in Romania, that have tried to characterize this monofloral honey [21,47,85,86], but none of them have characterized FJH in comparison with its origin source plant.

Thus, it is interesting to observe to what degree the bioactive compounds found in the FJ plant are transferred into its related honey, as the potential of FJH and its possible use in medicinal fields remains currently unexplored.

**Figure 1:**
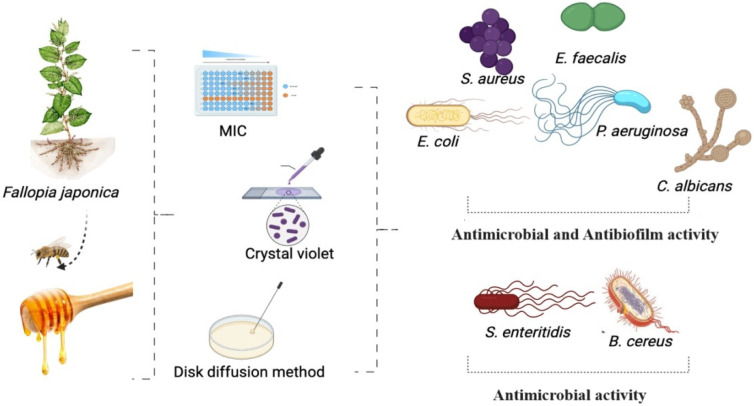
Antibacterial and antibiofilm activity of FJ plant and FJH (created with https://BioRender.com, accessed on 22 April 2024).

The aim of this study was as follows (Figure 1): (1) to identify, quantify, and compare, for the first time, the phenolic compounds of both FJ plants (different tissues) and honey, from three different parts of Romania; (2) to investigate and compare the antibacterial and antibiofilm potential of both plant tissues and honey samples; (3) to assess the contribution of different individual bioactive compounds to the overall antimicrobial and antibiofilm potential of the tested samples.

## 2. Materials and Methods

### 2.1. Plant and Honey Collection and Preparation

The plant raw material (roots, rhizomes, stems, and leaves of FJ plants) was collected in May–June 2023, from riparian ecosystems situated in the agricultural area of three experimental sites, located in the north-western part of Romania, namely Merișor (Maramureș county 47°39′25.2′′ N 23°24′06.7′′ E) and Valea Vinului (Satu Mare county 47°43′46.9′′ N 23°10′26.3′′ E), and the western part of Romania, namely Bocsig (Arad county (46°25′50.4′′ N 21°57′47.3′′ E), respectively, as previously reported in another study [21].

The plant tissues were air-dried in the dark at room temperature for one week, then ground using a laboratory mill and finally sealed in bags and kept at 4 °C until analysis [21].

The honey samples were acquired directly from local beekeepers that carried out pastoral beekeeping in the same experimental sites from where the plants were collected. From each experimental site, three different samples were purchased, in November 2022, a few months after their production (the honey is produced in September, as FJ is one of the latest-blooming nectariferous plants) [21]. These samples were stored in glass containers and kept at a temperature of 5 °C until further analyses were carried out [21].

For the preparation of the samples, 1 g of each sample of plant material (roots, rhizomes, stems and leaves of FJ plant) was individually extracted three times with 50 mL of methanol–water (1:1 *v*/*v*) pH = 2 (adjusted with HCl) at room temperature for 24 h after being sonicated (Bandelin Sonorex, Berlin, Germany) for 1 h. The supernatants were collected and evaporated to a final volume of 50 mL in a rotary evaporator (Buchi, Labortechnik, Falwil, AG Switzerland) and subsequently stored until analysis (4 °C). For determining the antimicrobial activity of the plant material, 1 g of each sample (roots, rhizomes, stems, and leaves of FJ plants) was individually extracted three times with 5 mL of ethanol–water (70:20, *v*/*v*) at room temperature for 24 h after being sonicated for 1 h. The supernatants were collected and evaporated. After that, the samples were reconstituted in 10 mL of ultrapure water to obtain a stock solution of 100 mg/mL. Before use, the supernatants were filtered through 0.22 µm filters. Honey samples were subjected to liquid–liquid extraction to remove the sugars and to release the phenolics entities. Ten grams of honey were diluted with acidified water (pH = 2 with concentrated HCl) and homogenized until complete dissolution. The mixture was placed into a separation funnel and extracted three times with ethyl acetate. All extracts were reunited, evaporated to dryness and stored until further analysis. Upon analysis, dried extracts were redissolved in HPLC grade methanol (10 mL), filtered through 0.45 µm nylon Millipore membranes and injected into the HPLC equipment (10 µL). To assess the antimicrobial activity of the honey, a weighted amount of each honey sample (approx. 1 g) was diluted in distilled water, in a 1:5 (*w*/*v*) ratio, obtaining a stock solution with a concentration of 200 mg/mL. Serial dilutions of honey samples were prepared aseptically for use in the agar well diffusion, MIC assay, and antibiofilm activity testing.

The analyses of both plant tissues and honey samples were performed at the Centre for Advanced Research and Extension in Apiculture (APHIS-DIA) in Cluj-Napoca, Romania.

The FJ honey samples were designated in the study as follows: FJH1-FJH3 (representing the samples from Merișor, Maramureș county); FJH4-FJH6 (representing the samples from Valea Vinului, Satu Mare county); and FJH7-FJH9 (representing the samples from Bocsig, Arad county).

### 2.2. Determination of Phenolic Compounds Using HPLC-PDA Method

HPLC analysis was conducted using the method proposed by Campos and Markham (2007) [87], with some modifications. The analysis was performed using a Shimadzu binary gradient unit LC-10AD VP system (Shimadzu Instruments, Kyoto, Japan), equipped with an SCL10AVP system controller, SPP-M20A Prominence Diode Array Detector, LC-10ADSP binary pumps, CTO-10AVP column oven, and SIL-10AF autosampler [88]. Separation was carried out on a Teknokroma Mediterranean Sea 18 column measuring 15 × 0.46 cm with an internal diameter of 5 µm, while maintaining a flow rate of 1 mL/min. The gradient comprised 2.5 pH water (adjusted with orthophosphoric acid) as solvent A and acetonitrile as solvent B. The initial gradient was 5% solvent B, which was increased to 9% B in 3 min (at minute 12 of separation); at minute 20, it was increased to 13% B; at minute 30, to 33% B; from minute 42 to 60, a linear gradient to 43% B was applied; at minute 65, it was increased to 90% B; at minute 70, to 100% B, and then gradually decreased to 5% B until minute 78. The column temperature was maintained at 24 °C using a CTO-10AVP column oven (Shimadzu Corporation, Kyoto, Japan), and the separation was monitored within the wavelength range of 220–400 nm [89]. Each compound’s identification was carried out by comparing its retention time and UV spectrum with those of authentic standards (gallic, protocatechuic, p-OH-benzoic, vanillic, caffeic, syringic, chlorogenic, ferulic, p-coumaric, and rosmarinic acid, as well as flavonoids including catechin, rutin, quercetin, quercitrin, isoquercitrin, vanillin, epicatechin, naringin. naringenin, kaempferol, apigenin, and stilbene resveratrol). Individual stock solutions of 1000 mg/L in HPLC grade methanol were prepared. The individual calibration curves of the polyphenol standards were assembled in the range of 0.05–100 mg/L, by processing the signals recorded at the maximum wavelength of each standard, according to their intensity (Supplementary Table S1). All r^2^ values were higher than 0.995. Each phenolic product’s quantification was carried out according to the samples’ peak areas and the corresponding calibration curve of individual phenolic. Results were expressed as mg/kg matrix (ppm).

### 2.3. Antimicrobial Activity

The antimicrobial activity of samples was evaluated via the disk diffusion method and microdilution method [90,91]. The selected bacterial strains were the following: *Staphylococcus aureus* ATCC 25923 (*S. aureus*), *Bacillus cereus* ATCC 11778 (*B. cereus*), *Enterococcus faecalis* ATCC 29212 (*E. fecalis*), *Escherichia coli* ATCC 25922 (*E. coli*), *Pseudomonas aeruginosa* ATCC 27853 (*P. aeruginosa)*, *Salmonella enteritidis ATCC 13076* (*S. enteritidis*) and yeast, *Candida albicans ATCC 10231* (*C. albicans*).

#### 2.3.1. Disk Diffusion Method

A quantity of 0.5 mL of each microbial suspension was inoculated on Petri dishes with MH agar plates and SDA agar for *Candida* species after the suspensions were adjusted to a concentration of 0.5 McFarland [92]. After removing any leftover liquid, the agar surface was left to dry for 15 to 20 min at 35 °C. Following that, aseptic wells were formed and 20 µL of each sample, with a concentration of 100 mg/mL, was added to each well. For Gram-positive bacteria, a positive control amoxicillin disk (30 μg/mL) was employed, while for Gram-negative bacteria, a norfloxacin disk (10 μg/mL) was used. Additionally, for yeast, a miconazole disk (10 μg/mL) served as the positive control. Subsequently, the plates were incubated at 37 °C for 24 h for bacteria and at 28 °C for 48 h for the fungal strain. Following incubation, the diameters of the inhibition zones (in mm) were measured. The analysis was conducted in triplicate.

#### 2.3.2. Determination of the Minimum Inhibitory Concentrations (MICs)

MICs were determined using a serial microdilution technique in Mueller–Hinton broth supplemented according to species, with a final microorganism suspension of 0.5 McFarland [92]. Amoxicillin served as the positive control for Gram-positive bacteria, nor-floxacin for Gram-negative bacteria, and miconazole for yeast. Additionally, untreated bacteria were included as a negative control. Serial dilutions of honey samples and FJ plant extracts were prepared aseptically as follows: 50 mg/mL, 25 mg/mL, 12.5 mg/mL, 6.25 mg/mL, 3.12 mg/mL, 1.56 mg/mL, 0.78 mg/mL, 0.39 mg/mL, 0.19 mg/mL, 0.1 mg/mL, 0.05 mg/mL, and 0.02 mg/mL. Moreover, a control sugar solution (glucose, fructose, and sucrose) was analyzed at the same dilutions as the honey samples to exclude the possibility that the inhibitory effect could be attributed to the osmotic effect of sugar. Following 24 h of incubation at 37 °C, the plate was examined at 600 nm using a BioTek Synergy 2 multichannel spectrophotometer (BioTek Instruments, Winooski, VT, USA). The MIC for each microorganism was defined as the lowest concentration that exhibited 100% inhibition of microbial growth. The analysis was performed in triplicate.

### 2.4. Antibiofilm Activity

The antibiofilm activity of FJ plant extracts and honey extracts were evaluated using the crystal violet (CV) staining test. Mature biofilms of each evaluated bacterial strain were formed in sterile 96-well microplates, as previously described [93]. After incubation, non-adherent bacterial cells were removed, and the formed biofilms were treated with different concentrations (MIC, MIC × 2, and MIC × 4) of the studied extracts, which were prepared in brain–heart infusion (BHI) broth. After 24 h of incubation, biofilms were stained with 100 μL of CV for 30 min. Subsequently, plates were washed and air-dried prior to quantifying biofilm biomass using a microplate reader. The following formula determined the percentage of biofilm eradication: [(OD growth control-OD sample)/OD growth control] × 100 [94]. Untreated biofilms represented the negative controls.

### 2.5. Statistical Analysis

Analysis of variance was performed to identify statistically significant differences between the evaluated *Fallopia japonica* plant extracts and honey samples (*p* < 0.05). The statistical analyses were assessed using SPSS software (version 19). The data are represented as means ± standard deviation. Statistical difference between determinations was evaluated using one-way ANOVA. Utilizing the chorddiag package, diagrams were generated to assess the relationships and influence of the inhibitory activities of the evaluated honey and plant extracts. Using the R program (version 4.0.5), several packages, including cluster R, dendextend, and ggplot were utilized to generate heatmaps and dendrograms by means of the Euclidean distance method, with complete linkage, to highlight the discrepancies and similarities between the phenolic compounds and antimicrobial activities in the plant extracts and honey samples. Principal component analysis was employed to visualize trends associated with biofilm eradication after treatment with the plant tissues and honey extracts. A graphical representation of the correspondence of the evaluated datasets was visualized in a variable plot, wherein the interactions between different plant tissues and honey samples could be observed. This analysis provided valuable information on how these factors were interrelated in both plant and honey variants according to their area of collection.

## 3. Results

### 3.1. Screening and Quantification of Phenolic Compounds Found in FJ Plant Tissues Using HPLC–PDA

The bioactive compounds present in FJ plant tissues were identified and their relative contents across different anatomic parts were quantified (Table 1). The screening results showed the presence of 20 compounds, which included 8 phenolic acids (protocatechuic acid, p-hydroxybenzoic acid, vanillic acid, chlorogenic acid, caffeic acid, p-coumaric acid, ferulic acid, rosmarinic acid), 10 flavonoids (catechin, epicatechin, rutin, naringin, quercitrin, isoquercitrin, quercetin, kaempferol, naringenin, apigenin), one phenolic aldehyde (vanillin), and one stilbene (resveratrol).

The most notable were chlorogenic acid (92.83 ± 13.64 mg/kg stems from Valea Vinului to 5188.60 ± 85.86 mg/kg stems from Bocsig), ferulic acid (21.65 ± 11.45 mg/kg stems from Bocsig to 951.33 ± 45.67 mg/kg rhizomes from Valea Vinului), rutin (78.36 ± 23.56 mg/kg stems from Valea Vinului to 3042.48 ± 86.78 mg/kg leaves from Merișor), quercitrin (302.32 ± 23.56 mg/kg stems from Valea Vinului to 6480.95 ± 113.56 mg/kg leaves from Valea Vinului) and resveratrol (values ranging from 55.95 ± 29.64 mg/kg in stems from Valea Vinului to 596.05 ± 82.49 mg/kg in roots from Merișor). On the contrary, lower results were observed for protocatechuic, p-hydroxybenzoic, vanillic, and p-coumaric acids.

Higher and lower contents of individual polyphenols were found for the Bocsig area (Arad county) in the case of naringin (5513.05 ± 189.45 mg/kg) found in stem tissues and kaempferol (3.18 ± 0.73 mg/kg) in leaf tissues. Vanillic, p-coumaric and rosmarinic acids, together with naringin and naringenin, were found only in the stem tissues from the Bocsig area (Arad county). There were significant variations observed among all areas. Additionally, resveratrol was detected in nearly all FJ plant samples across the experimental sites, except in the leaves Valea Vinului (Satu Mare county), where it was not found. Among the experimental sites, the Bocsig area exhibited the highest quantity of polyphenolic compounds, followed by the Merișor area and lastly, the Valea Vinului area.

### 3.2. Screening and Quantification of Phenolic Compounds Found in FJH Using HPLC-PDA

The phenolic compounds of FJH samples are reported in Table 2. Compared with the content of polyphenolic compounds found in FJ plant tissues, in the case of FJH, we identified only 15 polyphenolic compounds, namely, nine phenolic acids (gallic acid, p-hydroxybenzoic acid, vanillic acid, chlorogenic acid, caffeic acid, syringic acid, p-coumaric acid, ferulic acid, rosmarinic acid), five flavonoids (quercitrin, quercetin, naringenin, kaempferol, galangin), and one stilbene (resveratrol). The greatest concentration was obtained for gallic acid (8.50 ± 2.01 mg/kg) in one of the samples from the Bocisg area (Arad county), whereas samples from the Merișor area (Maramureș county) showed the lowest concentration of flavonoids (galangin 0.06 ±0.00 mg/kg). Generally, high values of gallic, p-hydroxybenzoic acid, p-coumaric, and ferulic acids were present in all FJH samples. Rosmarinic acid was detected only in samples from the Merișor area (Maramureș county) and the Bocsig area (Arad county), while galangin was observed in higher concentration in samples from the Bocsig area (Arad county).

Resveratrol was identified in all examined samples, with the sole exception being one sample from Maramureș county (Merișor area). Important amounts of quercitrin, quercetin, and kaempferol were also distinguished in all FJH samples. Tests on samples from Arad county (Bocsig area) indicated the presence of a larger quantity of polyphenolic compounds compared with the other two experimental sites.

### 3.3. Antibacterial Activity

The antibacterial mechanisms of different FJ plant tissues and honey samples against common Gram-positive bacteria (such as *S. aureus*, *B. cereus*, *E. faecalis*) and Gram-negative bacteria (including *E. coli*, *P. aeruginosa*, *S. enteritidis*), as well as yeast (*C. albicans*), are extensively detailed in Table 3 and Table 4.

#### 3.3.1. Inhibition Zones of FJ Plant Extracts against Different Microorganisms

A significant spectrum of antibacterial effects of the extracts of FJ plant tissues was documented, varying according to the tested strain. Although the plant extracts showed sensitivity against both Gram-negative and Gram-positive strains, the Gram-negative bacteria exhibited, in general, a larger inhibition diameter for all the plant tissues from all three experimental sites (Table 3).

*E. coli* demonstrated the highest sensitivity to the FJ plant extracts, especially to roots; the sample from the Merișor area (Maramureș county) displayed the most significant inhibition diameter (27.75 ± 0.61 mm), followed by those from the Valea Vinului area (Satu Mare county) (26.08 ± 0.45 mm) and Bocsig area (Arad county) (24.50 ± 0.24 mm). Of all the Gram-negative bacterial strains tested, *P. aeruginosa* was the most resistant, showing the lowest inhibition diameter when treated with samples from the upper tissues of the FJ plant, with values ranging from 12.17 ± 0.33 mm (leaves) and 16.48 ± 0.35 mm (stems) for the Valea Vinului samples (Satu Mare county) to 14.72 ± 0.37 mm (leaves) and 15.86 ± 0.29 mm (stems) for the Merișor samples (Maramureș county). Regarding the Gram-positive strains, *S. aureus* was the most sensitive to all plant tissues, in comparison with *B. cereus* that showed the lowest resistance.

Concerning the impact of FJ plant tissues on *C. albicans*, the root extract from Valea Vinului (Satu Mare county) demonstrated the most favorable effect, with an inhibition diameter of 17.52 ± 0.35 mm, whereas the leaves from Bocsig (Maramureș county) showed a moderate effect and thus, a less significant inhibition zone of 8.52 ± 0.25 mm. Among all the experimental areas, Valea Vinului and Bocsig (Satu Mare and Arad counties) exhibited the most effective antibacterial activity against all tested microbial strains.

#### 3.3.2. Inhibition Zones of FJH Extracts against Different Microorganisms

The same bacterial strains used for testing the inhibition potential of FJ plant extracts from three different regions of Romania were also employed in the cases of FJH samples from the same geographical locations (Table 4).

The disk diffusion method revealed that FJH samples had higher antibacterial activity against Gram-positive *S. aureus*, moderate activity against Gram-negative *E. coli*, and a low effect on *Candida* species.

The growth inhibition zones ranged from 13.63 ± 0.41 mm to 21.56 ± 0.43 mm against *S. aureus*, while *E. coli* displayed an inhibition diameter extending from 8.26 ± 0.34 mm to 19.95 ± 0.52 mm. FJH samples from all three experimental sites manifested the ability to inhibit *C. albicans* within the range of 6.26 ± 0.50 mm to 14.54 ± 0.12 mm inhibition zones.

From all the experimental sites, the honey samples from Arad county exhibited the highest antimicrobial activity against all tested strains, followed by those from Satu Mare county and lastly, Maramureș county. However, the samples from Maramureș and Satu Mare counties were statistically similar in terms of their characteristics against the tested microorganisms and demonstrated a lower sensitivity against *B. cereus* compared with the Gram-negative strains (*E. coli*, *P. aeruginosa*, *S. enteritidis*), against which FJH samples displayed the most potent antimicrobial activity.

#### 3.3.3. Minimum Inhibitory Concentration (MIC) (µg/mL) of FJ Plant Extracts against Different Microorganisms

**Figure 2:**
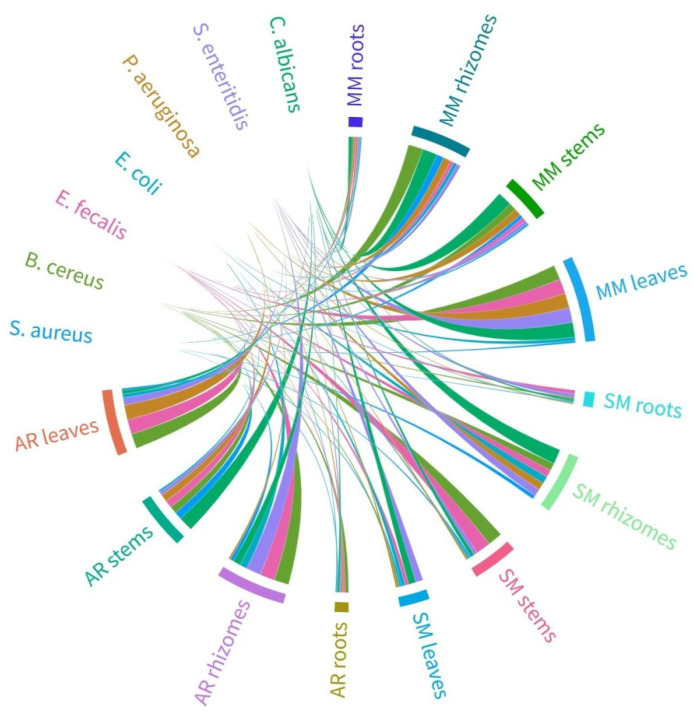
Chord diagram based on the antimicrobial potential (i.e., MIC) of the evaluated plant extracts against multiple microorganisms and according to their collection areas.

To ensure accuracy and precision in the results and to assess the extent to which the antimicrobial effect of the FJ plant extracts was transferred into the FJH samples, determination of the minimum inhibitory concentration (MIC) was incorporated alongside the disk diffusion method. The MIC of FJ plant extracts necessary to fully inhibit the microbial growth of the tested strains and the evaluated interconnection between plant extracts and bacterial strains is visualized in Figure 2. More details can be found in Supplementary Table S2. At the tested concentrations, the control sugar solutions (glucose, fructose, and sucrose) did not exhibit any inhibitory effects on microbial growth. This test was conducted to ensure that the antimicrobial activity observed in the honey samples was not due to osmotic effects but could be attributed to the intrinsic antimicrobial compounds present in the honey. As the control sugar solutions showed no inhibitory effect, they are not included in Figure 2.

The results from MIC testing revealed that the FJ plant extracts displayed inhibitory efficacy against Gram-negative bacteria, specifically *E. coli* and *P. aeruginosa*. Similar high MIC values were recorded for Gram-positive strains (*B. cereus* and *E. faecalis*) and yeast *C. albicans*, while *S. aureus* demonstrated the lowest MIC values for all plant tissue samples. Concerning the plant tissues, the most effective were the roots, followed by stems, whereas the most susceptible for microorganisms were the rhizomes and leaves. Valea Vinului (Satu Mare county) was the most potent area regarding the antibacterial potential of FJ plant tissues against the seven tested strains.

#### 3.3.4. Minimum Inhibitory Concentration (MIC) (µg/mL) of FJH Extracts against Different Microorganisms

**Figure 3:**
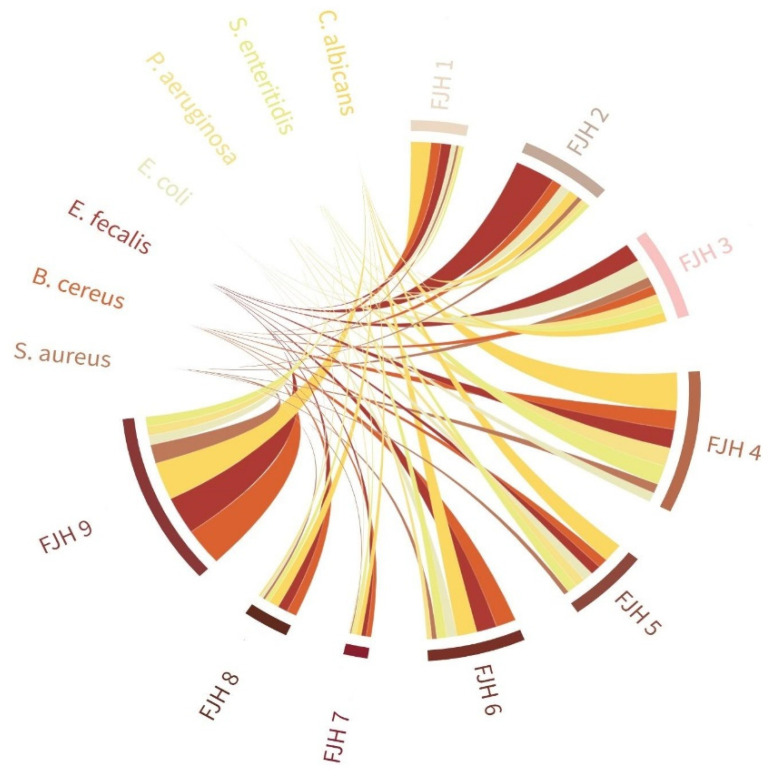
Chord diagram based on the interconnections between the antimicrobial potential (i.e., MIC) of the evaluated honey samples against multiple microorganisms according to their collection areas.

The minimum inhibitory concentration of nine FJH samples against seven species of pathogenic bacteria and their interconnections were also assessed (please refer to Figure 3 and Supplementary Table S3). The results revealed varying levels of effectiveness, with all samples demonstrating the highest inhibition of the Gram-negative bacteria, especially *E. coli*, especially FJH 7 samples from Arad county (Bocsig area). However, referring to FJH9, samples from the same county also exhibited the least effect against the tested Gram-positive pathogens, especially *B. cereus* and *E. faecalis*.

In contrast, the honey samples from Maramureș county (Merișor area) were the most effective against Gram-negative strains, especially *P*. *aeruginosa* and *S. enteritidis* (FJH 1 and FJH 2). In the analyzed FJH samples, Gram-negative bacteria typically exhibited higher resistance compared with Gram-positive bacteria.

### 3.4. Antibiofilm Activity

**Figure 4:**
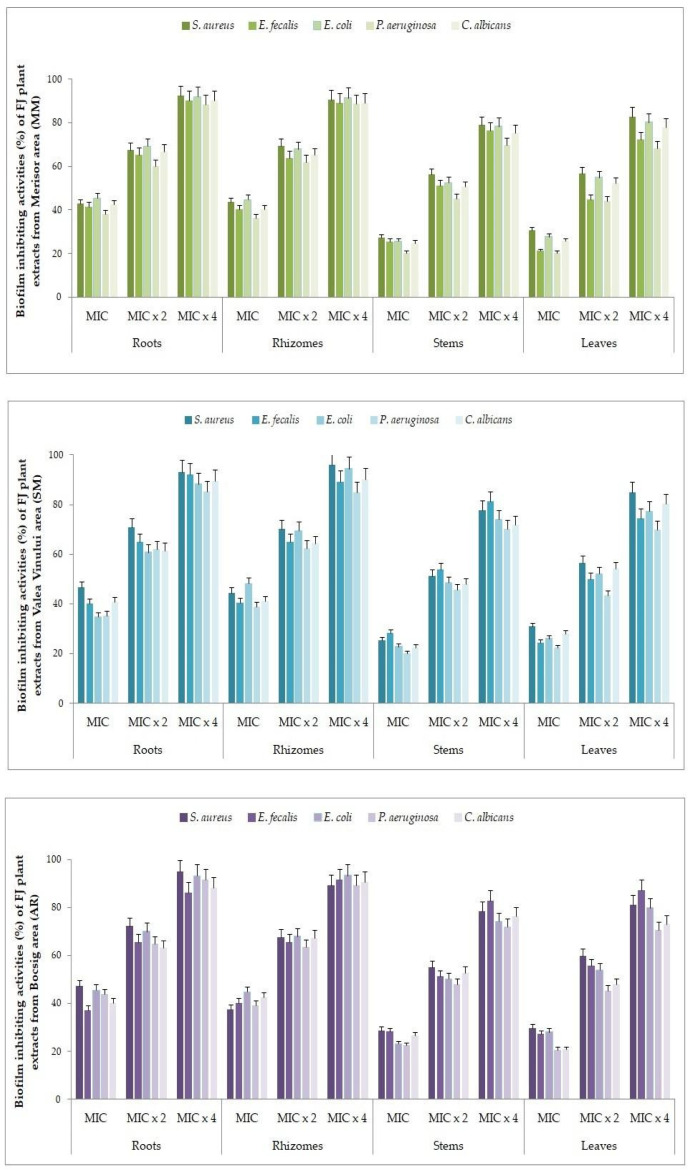
Percentages of biofilm eradication after treatment with various concentrations of FJ plant extracts.

**Figure 5:**
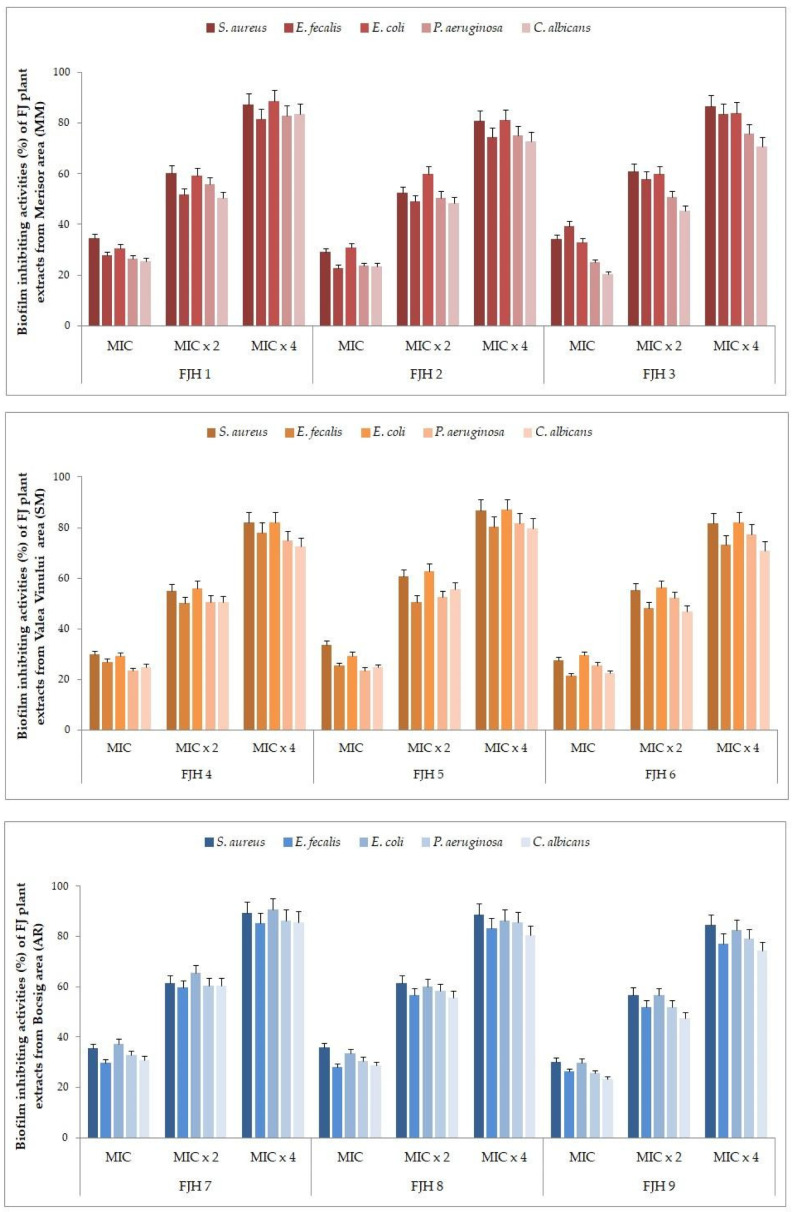
Percentages of biofilm eradication after treatment with various concentrations of FJH extracts.

The antibiofilm activity of both FJ plant extracts and FJH samples is presented in Figure 4 and Figure 5. The most common biofilm-forming bacterial strains (*S. aureus*, *E. faecalis*, *E. coli*, *P. aeruginosa*, *C. albicans*) were treated with extracts of FJ plant tissues and honey, respectively, at various concentrations (MIC, MIC × 2, MIC × 4).

#### 3.4.1. Biofilm Eradication after Treatment with Various Concentrations of FJ Plant Extracts

The FJ plant extracts from different tissues displayed diverse effects on the development of preformed biofilms, resulting in percentage reduction values ranging from 20.25 ± 0.56% (*P. aeruginosa*) to 95.98 ± 0.45% (*S. aureus*) (see Figure 4 and Supplementary Table S4). The strongest inhibition of biofilm formation was observed in *S. aureus*, for all four plant tissues extracts, followed by *E. coli*. The lowest efficacy of the plant extracts was found against *P. aeruginosa* and *E. faecalis*. Root and rhizome extracts manifested the greatest values for biofilm eradication, while the stems showed a reduced biofilm inhibition. The samples from Arad and Satu Mare counties (Bocsig and Valea Vinului areas) were the most efficient in biofilm eradication, followed by Maramureș county (Merișor area). Strains exposed to a concentration of MIC × 4 showed the highest biofilm eradication rates and the effect was determined to be dependent on the concentration of the extracts.

#### 3.4.2. Percentages of Biofilm Eradication after Treatment with Various Concentrations of FJH

Regarding the effect that the FJH samples exhibited related to the biofilm eradication of the same pathogens, the percentages are presented in Figure 5 (Supplementary Table S5). Notably, *S. aureus* and *E. coli* were the most sensitive to the FJH extracts, with the highest biofilm inhibition for the samples from Arad county and Maramureș county (Bocsig and Merișor areas), for MIC × 4 concentration. Among all bacteria, the biofilm formed by *E. faecalis* was the most resistant to the action of FJH, followed by that of the yeast *C. albicans*. The effect achieved was determined to be dependent on concentration, with the most significant reduction in the biofilm observed at the highest concentrations applied (MIC × 4).

### 3.5. Multivariate Statistics

**Figure 6:**
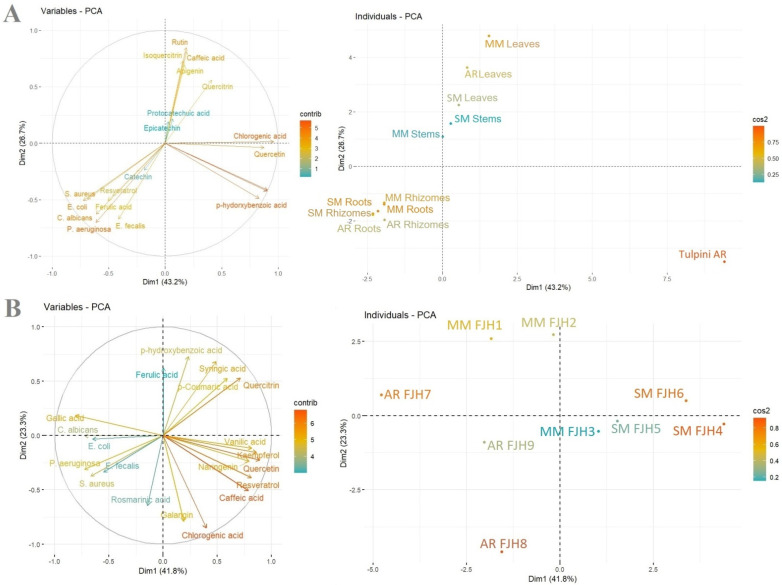
PCA visualizations of the evaluated plant tissue (**A**) and honey extracts (**B**) and the relationship between the biofilm eradication (MIC × 4) capability and the identified individual phenolic compounds. The first two dimensions of the plant extracts explained 69.9% of the total variance. Regarding the honey samples, the first dimensions explained 65.1% of the overall variance.

Both the plant tissue extracts and honey extracts revealed discrepancies with regard to their biofilm eradication capabilities as influenced by the accumulation of phenolic compounds (Figure 6A,B).

The upper first and second (Figure 6A) quadrants highlight the grouping pattern of the leaf extracts and the stem extracts from Merișor and Valea Vinului. With reference to the Merișor leaf tissues, it seems apparent that the biofilm eradications were proved to be influenced by the accumulation in these tissues of particular components including phenolics (i.e., caffeic acid) and flavonoids (i.e., apigenin, isoquercitrin, quercitrin, and rutin). Concerning the stem tissue samples from the Merișor and Valea Vinului areas, it was noted that the biofilm activity was determined by the presence of epicatechin and protocatechuic acids. The third quadrant highlights the stem tissues from the Bocsig area with elevated levels and complex phenolic matrix; however, the biofilm eradication proved to be moderate. As evidenced in the lower fourth quadrant, the accumulation of several compounds including catechin, ferulic acid, and resveratrol in the rhizome and root tissues seemed to have significantly influenced the biofilm-inhibitory activities.

Moving forward to the honey samples (Figure 6B), the grouping pattern was apparently impacted by the geographical origin. The FJH 7 and FJH 8 samples from the Bocsig area exhibited significant antibiofilm eradication capacities in all evaluated bacterial strains, as highlighted by their distinct position in the outer layer of the plotted chart. These samples accumulated significant levels of both gallic and rosmarinic acids. The samples from the Merișor area accumulated elevated levels not limited to phenolic acids, including ferulic, p-coumaric, p-hydroxybenzoic, and syringic acids, but also quercitrin. The third quadrant evidences the samples from Valea Vinului which accumulated noticeable levels of flavonoids, including stilbenes (i.e., resveratrol). The biofilm-inhibitory potential of the Valea Vinului samples was found to present similarities in terms of efficiency, as reflected in their relatively close positions in the quadrant.

**Figure 7:**
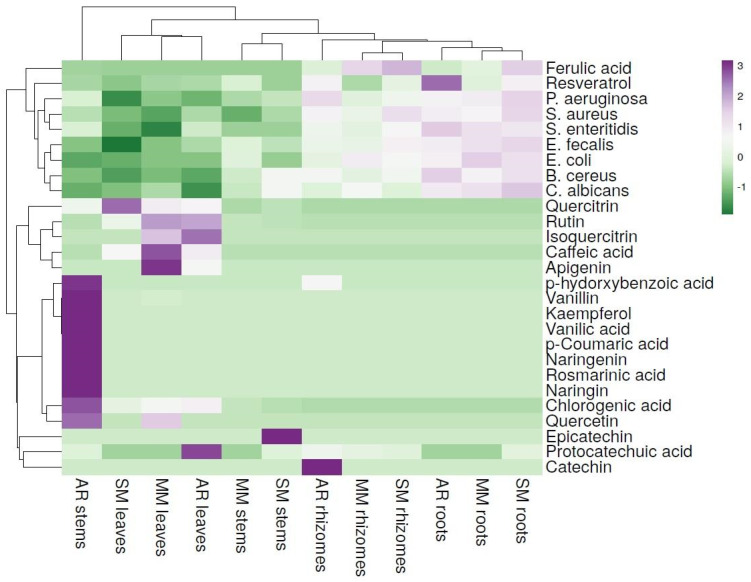
Heatmap visualization of the FJ plant extracts’ identified phenolic compounds and inhibitory activities against several microorganisms. Rows represent the individual phenolic compounds and the bacterial strains, and columns indicate the evaluated plant tissues according to their area of collection. The cells are highlighted according to the influence of both phenolic components and microorganisms, where an intense purple hue denotes a significant positive association and an intense green hue denotes a significant negative association.

**Figure 8:**
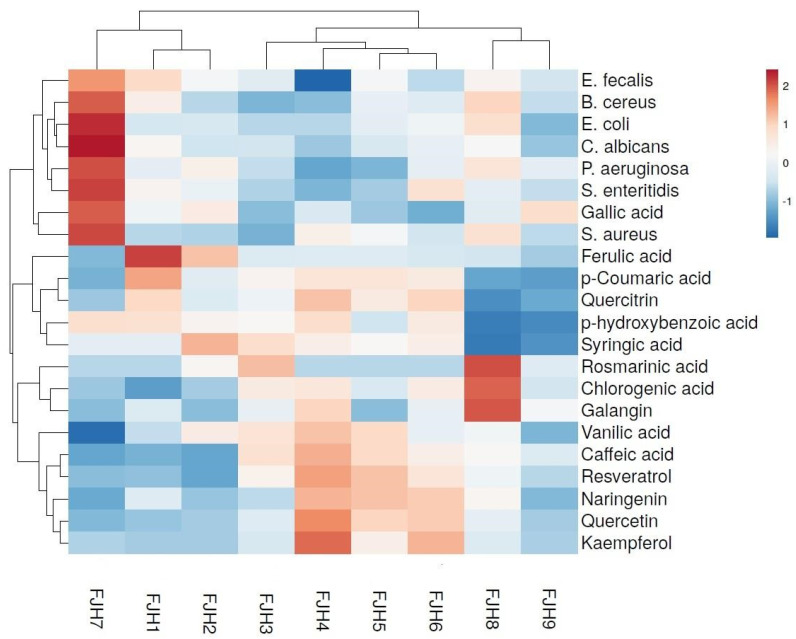
Heatmap visualization of the FJ honey extracts’ identified phenolic compounds and inhibitory activities against the evaluated bacterial strains. Rows represent the individual phenolic compounds and the microorganisms, and columns indicate the evaluated honey samples according to their area of collection. The cells are highlighted according to the influence of both phenolic components and microorganisms, where an intense red hue denotes a significant positive association and an intense blue hue denotes a significant negative association.

To fully comprehend and confirm the computed chord diagrams and PCA and clearly differentiate the evaluated plant samples according to their antimicrobial and antibiofilm capacities, HCA was conducted and heat maps were constructed (Figure 7 and Figure 8).

Regarding the plant extracts, a clear discrimination between the plant part and region of collection was observed. In Figure 7, the first cluster highlights the accumulation of elevated levels of phenolic acids (i.e., chlorogenic acid, p-hydroxybenzoic acid, and rosmarinic acid), phenolic aldehydes (i.e., vanillin), and flavonoids (i.e., naringin, naringenin, and kaempferol) in the stems of the plants collected from the Bocsig area, which presented moderate inhibitory activities, particularly against *S. aureus*, *S. enteritidis*, and *P. aeruginosa*.

Following the clustering of the dataset, the leaf extracts of the FJ plants were grouped together mainly due to their similar levels of accumulated phenolic acids (i.e., chlorogenic and caffeic acids) and flavonoids (i.e., rutin, and quercitrin), as well as apigenin and vanillin. Among the evaluated leaf samples, a relatively moderate inhibitory potential was observed against *E. coli*, *S. aureus*, and *S. enteritidis*, particularly from the samples collected from Arad county (Bocsig area). The subsequent sub-clustering emphasized the grouping of the stems extracts from the Valea Vinului and Merișor areas. The extracts from the stems accumulated similar levels of both chlorogenic acid and rutin, as well as epicatechin (solely in the samples from Satu Mare), and exhibited similarities in terms of inhibition against several bacterial strains including *S. aureus*, *E. coli*, *E. faecalis*, and *P. aeruginosa*. Subsequently, although the rhizomes did not significantly accumulate multiple phenolic compounds (i.e., ferulic acid, protocatechuic acid, catechin, and resveratrol), a significantly increased antimicrobial capacity was observed. Samples that accumulated resveratrol presented relatively high inhibitory activity against the evaluated microorganisms, suggesting its antibacterial potential, as previously demonstrated [95,96]. Particularly, the highest inhibitory activity was highlighted in the cases of *S. aureus* and *E. coli*, followed by increased activity against *S. enteritidis* and *P. aeruginosa*. The final sub-cluster included the root extracts with the highest inhibitory activities among the evaluated plant parts. Comparisons between the accumulation of phenolic compounds in the roots and rhizomes were recorded (i.e., ferulic acid, catechin, and resveratrol), which may suggest their strong inhibitory potential against all evaluated microorganisms.

Regarding the honey samples (Figure 8), one sample from Bocsig (i.e., FJH7) and two from Merișor (i.e., FJH1and FJH2) proved to have distinct accumulations in phenolic compounds compared with the other honey samples under study.

The sample collected from the Bocsig area (FJH7) revealed elevated levels of gallic acid and p-hydroxybenzoic acid, as indicated by the positive values highlighted according to the importance score. Interestingly, although phenolic acids were found to accumulate in rather scarce amounts in that sample, it presented significantly high inhibitory activity against all tested bacterial strains. In terms of effectiveness, FJH7 was followed by samples from the Merișor area (FJH1 and 2), which exhibited strong inhibitory capacities, notably with regard to *S. aureus*, *E. faecalis*, *P. aeruginosa*, and *S. enteritidis*. Subsequently, the remaining sample from Merișor (i.e., MM FJH3) was positioned collectively in the same sub-cluster with the samples from Valea Vinului (i.e., FJH4-6). These samples presented similarities in the accumulation of certain phenolic components (i.e., caffeic acid, chlorogenic acid, syringic acid, vanillic acid, and resveratrol). Furthermore, samples from Valea Vinului accumulated considerable build-up of certain flavonoids (i.e., kaempferol, naringenin, quercetin, and quercitrin). Surprisingly, despite the fact that the sample FJH4 displayed a considerable accumulation of relevant phenolic constituents, it demonstrated significantly reduced antimicrobial activity, particularly against *E. faecalis*. Interestingly, resveratrol was identified in all FJH samples, with higher amounts in those collected from Valea Vinului, signifying the use of resveratrol as a biomarker to identify the botanical origin of this type of honey and also its antibacterial activity. The subsequent sub-cluster grouped the other samples from the Bocsig area, namely FJH8 and FJH9, respectively. Sample FJH8 accumulated elevated levels of galangin, rosmarinic acid, and chlorogenic acid and presented inhibitory activities against both Gram-positive (i.e, *B. cereus* and *S. aureus*) and Gram-negative (*E. coli*, and *P. aeruginosa*) bacteria. Overall, as evidenced in the first cluster of sample FJH7, the samples from the Bocsig area presented increased antimicrobial activity against the tested bacterial strains (especially FJH8).

## 4. Discussion

### 4.1. Phenolic Compounds Found in FJ Plant and Honey

In the present study, the metabolic profiles of six different tissues: roots, rhizomes, stems, and leaves of FJ plants, along with nine different FJH samples, from different geographical regions of Romania, were determined using the HPLC-PDA method.

Our research identified chlorogenic acid in the same plant tissues as the study by Mikulic-Petkovsek et al., namely, the upper parts of the plants (shoots and leaves), but in our case, the values were higher than those presented by the above-mentioned authors [97]. The results recorded by Lachowicz et al. for chlorogenic acid (5-O-caffeoylquinic acid) in the same plant tissues (leaves and stalks) were significantly lower (30 ± 0.01 mg/kg leaves and 80 ± 0.00 mg/kg stalks) [98] compared with our study. For another identified flavonoid, rutin, the same authors reported results that were again lower than those for our three experimental areas. Thus, Mikulic-Petkovsek et al. reported a content of 85.70 ± 12.42 µg/kg rutin in the upper parts of the plant [97], while Lachowicz et al. observed a content of 30 ± 0.00 mg/kg in leaves and 20 ± 0.00 mg/kg in stems [98]. As for both quercitrin and isoquercitrin, the data values were again lower than our findings. The same studies did not identify quercetin in the upper parts of the plant, while our study observed the presence of this compound in the leaf tissue from the Merișor area (Maramureș county) and the stem tissue from the Bocsig area (Arad county). Our results for resveratrol were lower than those reported by Kim et al. [43] for the roots of *Polygonum cuspidatum* from China (2.90 mg/g), by Yi et al. [99] or Nawrot-Hadzik et al. [100] for rhizomes, or by Mikulic-Petkovsek et al. for the upper parts of the plant [98], but higher than those identified by Lachowicz et al. in stems and leaves [98]. Regarding the presence of the flavonoids epicatechin and catechin, our findings showed the presence of epicatechin only in the stem tissues from Satu Mare county and catechin only in the rhizome tissue from Maramureș and Arad counties, while Lachowicz et al. [98], Bensa et al. [38], and Mikulic-Petkovsek et al. [97] identified these compounds only in the upper parts of the FJ plant. However, Vrchotová et al. reported catechin and epicatechin to be present in the rhizomes and young sprouts of *Reynoutria japonica* plants from the Czech Republic [101], but in lower content.

The other compounds identified in our research in the plant tissues of FJ plants, such as p-hydroxybenzoic acid, vanillic acid, rosmarinic acid, naringin, or vanillin, were not observed in any other study that investigated the composition of FJ plant species.

We used multivariate analysis techniques to depict similarities and discrepancies between the different plant parts of *F. japonica* and the identified phenolic compounds. The rhizomes and roots of knotweed accumulated elevated levels of resveratrol and ferulic acid, which correlated with the inhibitory activities. Other studies clearly distinguished *Fallopia* sp. based on their morphological characteristics (i.e., leaves, stalks, and roots), phytochemical composition, and bioactivities, through the use of multivariate analysis (i.e., PCA) (Lachowicz et al., 2019) [99].

As for the phenolic compounds found in the honey samples, it is important to mention that these compounds are highly related to the botanical source of the plants visited by bees and transferred into the honey through the nectar [102]. Studies have shown that these compounds play a pivotal role in conferring honey’s health-promoting effects, especially antioxidative and antibacterial properties [103,104,105]. The most predominant phenolic compounds that can be found in honey are phenolic acids (such as gallic acid, p-coumaric acid, ferulic acid, caffeic acid, benzoic acid, vanillic acid, cinnamic acid) [106] and flavonoids (the most common being quercetin, rutin, galangin, kaempferol, luteolin, chrysin, apigenin, catechin, hesperetin, and naringenin) [107].

Some of these above-mentioned compounds can be identified as potential markers for classification and authentication of different types of honey, particularly monofloral varieties [108,109]. However, identifying these marker compounds in honey poses a challenge due to their low concentrations. Successful isolation and chemical identification heavily rely on the extraction and analysis methods utilized [110,111,112].

Unfortunately, there have been no other studies of FJH regarding its individual polyphenols, but as FJH shares the same botanical origin as buckwheat honey, we compared the reported polyphenolic compounds found in buckwheat honey to those identified in our study. In this regard, the study carried out by Nešović et al. [113] revealed the presence of 31 phenolic compounds found in buckwheat honey from Serbia and Poland, among them p-coumaric acid, p-hydroxybenzoic acid, caffeic acid, quercetin, kaempferol, galangin, and ferulic acid. Contrary to the observations made in our study, gallic acid was not detected in any of the honey samples from Serbia or Poland. Overall, the values obtained by those authors were higher than those presented in our manuscript [113]. Likewise, Socha et al. [114] documented high concentrations of phenolic acids in buckwheat honey, particularly gallic (913.79 ± 37.97 µg/100 g) and caffeic (707.21 ± 32.4 µg/100 g) acids, together with p-coumaric (360.15 ± 1.0 µg/100 g), ferulic (54.13 ± 1.6 µg/100 g), and syringic acid (47.68 ± 3.2 µg/100 g). These values were much lower than our findings. However, our study showed higher results for caffeic acid, p-coumaric, syringic, and ferulic acids compared with the data reported by Kędzierska-Matysek et al. for Polish buckwheat honey samples [115]. Vanillic acid was also reported by Puścion-Jakubik et al. [116] for a variety of Polish honeys, including buckwheat honey, but their results were lower than our findings.

Of all the polyphenolic compounds identified in FJH, the most worthy of mention is resveratrol, as this is the first attempt to demonstrate the presence of this bioactive compound and its transfer from FJ plant to FJH. As resveratrol was identified in eight out of the nine honey samples, it may potentially be considered as a biomarker for the botanical origin of FJH. Another honey that contains resveratrol in its composition is sage honey, as demonstrated by Gašić et al. [117].

### 4.2. Antimicrobial Effect of FJ Plant and Honey

Based on the results previously reported by researchers [69,98,118], the rich content of bioactive substances such as polyphenols could influence the antibacterial mechanism of the FJ plant, suppressing the hydrolytic enzymes or other interactions that deactivate the microbial adhesion proteins, resulting in the disruption of bacterial cell growth [19]. The antibacterial activity of FJ plant tissues was strongly associated with the content of phenolic compounds, as all the root samples of FJ plants had the highest content of individual polyphenols, thus showing the best antimicrobial effect. Other studies have confirmed the same trend [65,119,120]. Shan et al. tested crude extract of *Reynoutria japonica* roots on five foodborne bacteria (*B. cereus*, *Listeria monocytogenes*, *S. aureus*, *E. coli*, and *Salmonella anatum*) showing that the strong antibacterial properties, especially in the case of *S. aureus* and *B. cereus*, were due to stilbenes (mainly piceid) and hydroxyanthraquinones (mainly emodin) [65]. Their results were not the same as our findings, as our inhibition diameters for the root extracts were higher (18.54 ± 0.37 mm for *B. cereus*, 28.05 ± 0.22 mm for *S. aureus*), while the MIC values were lower (0.39 µg/mL *B. cereus* and 0.19 µg/mL for *S. aureus*). Moreover, in our study, *E. coli* had higher sensitivity than *B. cereus* to the root extract [65]. On the other hand, Su et al. confirmed that root extracts of *Polygonum cuspidatum* manifested antibacterial activity against the tested strains of *S. aureus* and *P. aeruginosa*, but with an average inhibition zone smaller than the results presented in our study (Table 3). For the MIC, our results were similar in the case of *S. aureus*, but were lower for *P. aerugionsa* [19]. Testing the same *Polygonum cuspidatum* tissue (root), Wang et al. [120] observed that the main phenolic higher alcohol found in the root tissue was phenethyl alcohol. However, the plant extract did not have any inhibitory effect against *E. coli* or *C. albicans*; the only strain sensitive to the root extract was the Gram-positive bacteria *S. aureus*, with half of the inhibition diameter depicted in our study (namely 12.4 ± 0.1 mm, compared with our results that ranged from 24.54 ± 0.35 mm to 28.05 ± 0.22 mm) [120]. Contrary to this study, Zhang et al. demonstrated that *Reynoutria japonica* plant extract (not mentioning exactly which tissue of the plant was used) was able to inhibit the growth of the yeast *C. albicans* [39]. However, the results were lower than those shown in our study. Piceid, resveratrol, and emodin were considered the main active compounds that influenced the inhibition of bacterial growth on *Polygonum cuspidatum* plant extract in a study carried out by Liu et al. [121]. That study demonstrated that MIC values for the rhizome extracts of the plant were not detected against the *E. coli* strain, and the values were higher against *S. aureus* (50 μg/mL) and *P. aeruginosa* (100 μg/mL) compared with our findings (1.56 μg/mL and 3.12 μg/mL, respectively). The differences between our results and those of other authors may be due to the use of different bacterial strains or varying methods and conditions for cultivating the bacteria. These factors can significantly influence the observed antimicrobial effects, leading to variations in the results.

As can be observed, the highest inhibition by all FJ plant extracts was demonstrated against the *S. aureus* bacterial strain. This can be explained through the content of resveratrol found in the plant extracts, as this compound exhibits protective effects against *S. aureus* infections and suppresses the quorum-sensing ability of this bacterium by targeting its infectious proteins [122].

However, the antibacterial efficacy of different plant tissues of FJ depends on the concentrations of different bioactive compounds, as some of these compounds are found in the underground parts of the plant and are strongly influenced by the soil characteristics, temperature, and the growth stage of the plant [123]. This may explain the differences in quantities of bioactive compounds and antibacterial properties for the aboveground parts of our FJ plant extracts, as our samples were collected in May–June, the growing season of the plant, when not all the tissue had yet reached full maturity.

Nevertheless, despite the high territorial coverage of FJ plant species worldwide and its floral abundance and beekeeping potential, the antimicrobial properties of its related honey have not been adequately investigated. To the best of our knowledge, no other study has been published on the comparative antibacterial effect of FJ plant tissues and FJH. In addition, no other research has so far assessed the antibiofilm activity of both the FJ plant and its related honey.

The antibacterial potency of honey, and more specifically of monofloral honey, is generally associated with the geographical and botanical origin of the raw material [109,124], as the content of polyphenols can influence its beneficial health effects [104,125,126], based on their ability to reduce antibiotic resistance in infectious bacteria and inhibit biofilm formation [81,127]. Since ancient times, the antimicrobial efficacy of honey has been extensively evaluated in order to gather supporting evidence for its utilization as a natural remedy [128,129,130].

Our findings on the FJH’s antibacterial properties demonstrate that the bioactive compounds and the inhibition characteristics of the raw plant are transferred to the honey. Our findings suggest a general moderate inhibition effect (<15 mm) in the FJH samples investigated in this study, compared with the values obtained for FJ plant extracts. Even if the antibacterial potential of FJH is less significant than that of the FJ plant in terms of the inhibition zones and minimum inhibitory concentration, the results are still noteworthy compared with other monofloral honeys that are known to have powerful antibacterial properties, such as buckwheat or manuka [112,131,132,133,134,135].

The results presented in our findings are quite different from those presented by Pătruică et al. [86], who evaluated for the first time the antimicrobial effect of FJH in terms of bacterial inhibition rate (BIR%) and mycelial inhibition rate (MIR%). The tested strains were *Streptococcus pyogenes*, *Streptococcus aureus*, *Shigella flexneri*, *Pseudomonas aeruginosa*, *Escherichia coli*, *Salmonella typhimurium*, *Haemophilus influenzae* type B, *Listeria monocytogenes*, *Bacillus cereus*, *Candida albicans*, and *Candida parapsilosis*. In our study, the best inhibition results for FJH were against *S. aureus*, followed by *E. coli* and *P. aeruginosa*, while the study by Pătruică et al. showed the best inhibition against *E. coli* (26.93%), *B. cereus* (21.28%), and *C. albicans* (44.61%) [86].

The antimicrobial effect of FJH was largely due to the presence of resveratrol in almost all the honey samples, with values ranging from 0.168 ± 0.011 mg/kg to 1.686 ± 0.0763 mg/kg, except one sample from the Merișor area where resveratrol was not detected. A higher content of resveratrol was found in samples from the Valea Vinului area, thus correlating with the antibacterial potential of the plant and honey samples from this area. Other important compounds found in large quantities in all the analyzed honey samples may have influenced the antimicrobial and antibiofilm activity. Thus, gallic acid [136], p-coumaric acid [137], p-hydroxybenzoic acid [138], or ferulic acid [139] can also be considered as possibly responsible for the beneficial health effects of FJH.

### 4.3. Antibiofilm Activity of FJ Plant and Honey

These plant-derived compounds have demonstrated synergistic effects when combined with conventional antibiotics [140]. Phenolic compounds like quercetin [141], epigallocatechin-3-gallate [142], rutin [143], and especially resveratrol [144,145] extracted from FJ plant tissues (roots or rhizomes) play a crucial role in enhancing antibiotic efficacy against resistant pathogens, such as *S. aureus*, *P. aeruginosa*, *E. coli*, *Streptococcus mutans*, or *C. albicans*.

The study of FJ plant extracts reveals their potential as natural antibiofilm agents, particularly against *S. aureus* biofilms, where they demonstrated up to 95.98% reduction in biofilm formation. However, their effectiveness varied across different bacterial species and was notably lower against *P. aeruginosa* and *E. faecalis*, which are known for robust biofilm formation. Variations in antibiofilm activity results can be caused by different bacterial strains within the same species or by different conditions in the cultivation environment. Furthermore, differences in antibiofilm activity were also influenced by the plant tissue type, with roots and rhizomes showing the most potent effects, suggesting a higher concentration of bioactive compounds in these parts compared with the stems. Additionally, environmental factors seemed to affect the efficacy, as extracts from different geographical locations showed varying degrees of biofilm eradication. Similar results were published by Zeng et al. regarding the suppression of the formation of *S. aureus* biofilms by *Polygonum chinense* L. aqueous extracts [146]. In that case, the antimicrobial and antibiofilm properties of *Polygonum chinense* L. were attributed to gallic acid, a compound found in FJ plants that is able to disrupt the bacterial cell wall structure of *S. aureus* and hamper the synthesis process, leading to inefficiency [147].

The antibiofilm activity of FJH samples was particularly effective against *S. aureus* and *E. coli*, especially in samples from Arad and Maramureș counties (Bocsig and Merișor areas) at high concentrations (MIC × 4). The effectiveness of FJH varied, with *E. faecalis* and *C. albicans* biofilms showing more resistance, highlighting a limitation in the antibiofilm spectrum of the honey extracts. Unfortunately, to the best of our knowledge, there are currently no available data describing the antibiofilm potential of this type of honey. Nonetheless, certain studies have revealed the potential of buckwheat honey, derived from the same *Polygonaceae* family as *Fallopia japonica*, to eradicate biofilms formed by *Streptococcus mutans* [148]. The same research revealed that buckwheat honey demonstrated antibiofilm properties similar to those of manuka honey [148]. Another study, carried out on Latvian honeys, including buckwheat honey, confirmed the antibiofilm capacity of the latter against the *E. coli* bacterial strain [149], while Polish buckwheat honey demonstrated effectiveness against staphylococci present in biofilm structures [150].

The concentration-dependent nature of biofilm eradication by FJH indicates that high concentrations are necessary for significant biofilm control. Further research is needed to optimize the dosing and broaden the effectiveness of FJH against a wider range of biofilm-forming organisms.

Both FJ plant extracts and FJH samples demonstrated promising antibiofilm activity, with each showing variable effectiveness against different pathogens. FJ plant extracts were most potent against *S. aureus*, displaying their potential in targeting bacterial biofilms. FJH samples also showed good antibiofilm activity, especially against *S. aureus* and *E. coli*, but were less effective against *E. faecalis* and yeast *C. albicans*, indicating specific limitations in their antibiofilm spectrum. The concentration of active compounds plays a critical role in both types of extracts, with higher concentrations generally yielding better results. This suggests that optimizing the concentration and delivery methods could enhance the antibiofilm efficacy of both FJ plant extracts and FJH. Further comparative studies could help delineate the distinct mechanisms and optimize their applications in clinical settings.

## 5. Conclusions

In this study, we identified 20 phenolic compounds from the FJ plant and 15 compounds from FJH, using the HPLC-PDA method. The most notable compounds from FJ plant tissues were ferulic acid, rosmarinic acid, chlorogenic acid, and protocatechuic acid, along with the flavonoids naringin, naringenin, kaempferol, and quercitrin. The phenolic aldehyde vanillin and the stilbene resveratrol were also identified in considerable amounts. However, in FJH, the presence of various substances such as gallic, p-hydroxybenzoic, p-coumaric, and ferulic acids was also detected. Resveratrol was detected in almost all the honey samples, being considered a marker for the identification of FJH. Samples from the Bocsig area (Arad county) exhibited larger amounts of phenolic compounds, followed by those from the Merișor and Valea Vinului areas (Maramureș and Satu Mare counties). The inhibitory activity was evaluated through both the disk diffusion method and the microdilution method, while antibiofilm activity was assessed using the crystal violet staining test. The findings highlighted significant inhibition of FJ plant tissues and honey, particularly against Gram-negative bacterial strains, especially *E. coli*. For both FJ plant tissues and FJH, the strongest inhibition of biofilm formation was observed in *S. aureus* and *E. coli*, while the lowest efficacy was observed for *E. faecalis*, followed by the yeast *C. albicans*. In terms of the experimental sites, samples from both Arad and Satu Mare counties (Bocsig and Valea Vinului) exhibited the highest efficacy in antimicrobial inhibition and biofilm eradication, followed by samples from Maramureș county (Merișor area).

Multivariate statistical analysis was employed to correlate the presence of polyphenolic compounds in both FJ plant tissues and FJH samples and their influence on antimicrobial and antibiofilm activities. The findings indicated that rhizomes and roots of FJ plant had the most noticeable inhibitory activity in the tested bacterial strains due to the presence of ferulic acid, protocatechuic acid, catechin, and resveratrol. Moreover, in case of FJH samples, gallic, ferulic, vanillic, and caffeic acids together with resveratrol, kaempferol, quercetin, and naringenin were the most notable compounds that influenced the inhibition activity of the honey samples. However, distinct accumulations of phenolic compounds were observed depending on the geographical origin of the samples.

To the best of our knowledge, no study has previously been undertaken to assess the individual phenolic compounds found in both the FJ plant and its related honey. In addition, no other research has comparatively evaluated the antibacterial and antibiofilm properties of the FJ plant to those of FJH.

Given the escalating significance of bacterial antibiotic resistance, the promising results of the overlay assay provide insights into the antibacterial activity of FJH. Consequently, this unique type of honey emerges as a promising alternative antibacterial agent. As our findings are preliminary, further studies should be undertaken in order to fully assess the bioactive compounds of FJH through different assays. In addition, the current research could represent a sustainable approach to counter harmful plant eradication methods and strengthen beekeeping efforts for enhancing the promotion, certification, and commercialization of Romanian monofloral *Fallopia japonica* honey.

## Figures and Tables

**Table 1:** Content of polyphenolic compounds identified in FJ plant tissues from three different areas of Romania (mg/kg).

Group	Compound	Merișor				Valea Vinului				Bocsig			
		Roots	Rhizomes	Stems	Leaves	Roots	Rhizomes	Stems	Leaves	Roots	Rhizomes	Stems	Leaves
Phenolic acids	protocatechuic acid	n.d.	20.92 ± 6.72 ^b^	n.d.	n.d.	18.53 ± 5.22 ^bc^	17.53 ± 2.375 ^bc^	16.15 ± 1.97 ^c^	n.d.	n.d.	26.60 ± 9.58 ^b^	16.47 ± 3.82 ^b^	90.85 ± 17.89 ^a^
	p-hydroxybenzoic acid	n.d.	n.d.	n.d.	n.d.	n.d.	n.d.	n.d.	n.d.	n.d.	5.75 ± 2.51 ^b^	18.85 ± 8.45 ^a^	n.d.
	vanillic acid	n.d.	n.d.	n.d.	n.d.	n.d.	n.d.	n.d.	n.d.	n.d.	n.d.	12.8 ± 6.44	n.d.
	chlorogenic acid	n.d.	n.d.	218.05 ± 15.34 ^e^	1677.05 ± 19.85 ^c^	n.d.	n.d.	92.83 ± 13.64 ^f^	1132.75 ± 31.57 ^d^	n.d.	n.d.	5188.60 ± 85.86 ^a^	2152.90 ± 68.59 ^b^
	caffeic acid	n.d.	n.d.	n.d.	153.11 ± 23.45 ^a^	n.d.	n.d.	n.d.	51.37 ± 13.45 ^b^	n.d.	n.d.	n.d.	65.58 ± 24.56 ^b^
	p-coumaric acid	n.d.	n.d.	n.d.	n.d.	n.d.	n.d.	n.d.	n.d.	n.d.	n.d.	14.75 ± 8.45	n.d.
	ferulic acid	289.58 ± 34.56 ^c^	809.75 ± 39.78 ^b^	n.d.	n.d.	834.75 ± 67.89 ^ab^	951.33 ± 45.67 ^a^	n.d.	n.d.	168.64 ± 27.83 ^d^	236.35 ± 56.78 ^c^	21.65 ± 11.45 ^e^	n.d.
	rosmarinic acid	n.d.	n.d.	n.d.	n.d.	n.d.	n.d.	n.d.	n.d.	n.d.	n.d.	4638.53 ± 89.45	n.d.
Flavonoids	catechin	n.d.	n.d.	n.d.	n.d.	n.d.	n.d.	n.d.	n.d.	n.d.	511.95 ± 56.87	n.d.	n.d.
	epicatechin	n.d.	n.d.	n.d.	n.d.	n.d.	n.d.	216.95 ± 61.45	n.d.	n.d.	n.d.	n.d.	n.d.
	rutin	n.d.	n.d.	162.52 ± 21.45 ^c^	3042.48 ± 86.78 ^a^	n.d.	n.d.	78.36 ± 23.56 ^d^	948.83 ± 45.67 ^b^	n.d.	n.d.	n.d.	2940.76 ± 73.64 ^a^
	naringin	n.d.	n.d.	n.d.	n.d.	n.d.	n.d.	n.d.	n.d.	n.d.	n.d.	5513.05 ± 189.45	n.d.
	quercitrin	n.d.	n.d.	n.d.	3030.25 ± 89.45 ^b^	n.d.	n.d.	302.32 ± 23.56 ^c^	6480.95 ± 113.56 ^a^	n.d.	n.d.	2081.31 ± 34.56 ^b^	2695.23 ± 78.94 ^b^
	isoquercitrin	n.d.	n.d.	n.d.	681.05 ± 78.34 ^b^	n.d.	n.d.	n.d.	n.d.	n.d.	n.d.	n.d.	964.15 ± 89.62 ^a^
	quercetin	n.d.	n.d.	n.d.	144.65 ± 45.70 ^b^	n.d.	n.d.	n.d.	n.d.	n.d.	n.d.	221.52 ± 23.45 ^a^	n.d.
	kaempferol	n.d.	n.d.	n.d.	n.d.	n.d.	n.d.	n.d.	n.d.	n.d.	n.d.	5504.65 ± 197.45 ^a^	3.18 ± 0.73 ^b^
	naringenin	n.d.	n.d.	n.d.	n.d.	n.d.	n.d.	n.d.	n.d.	n.d.	n.d.	5326.05 ± 128.31	n.d.
	apigenin	n.d.	n.d.	n.d.	141.15 ± 38.56 ^a^	n.d.	n.d.	n.d.	n.d.	n.d.	n.d.	n.d.	38.75 ± 9.08 ^b^
Phenolic aldehydes	vanillin	n.d.	n.d.	n.d.	77.15 ± 11.28 ^b^	n.d.	n.d.	n.d.	n.d.	n.d.	n.d.	4385.55 ± 108.77 ^a^	21.05 ± 7.41 ^c^
Stilbenes	resveratrol	320.55 ± 48.91 ^cd^	106.25 ± 30.61 ^e^	242.87 ± 52.62 ^d^	92.80 ± 21.88 ^e^	596.05 ± 82.49 ^b^	373.52 ± 23.81 ^c^	55.95 ± 29.64 ^f^	n.d.	1234.50 ± 55.29 ^a^	578.33 ± 76.25 ^b^	88.55 ± 12.67 ^ef^	111.35 ± 29.83 ^e^

The values are presented as the mean of three replications ± standard deviations. Values in the same row marked with different superscript lowercase letters indicate statistically significant differences between the identified phenolic compounds (determined by post hoc Tukey HSD test, *p* < 0.05); n.d.—non-detected.

**Table 2:** Content of polyphenolic compounds identified in FJH from three different areas of Romania (mg/kg).

Group	Compound	Merișor			Valea Vinului			Bocsig		
		FJH1	FJH2	FJH3	FJH4	FJH5	FJH6	FJH7	FJH8	FJH9
Phenolic acids	gallic acid	6.28 ± 1.09 ^b^	6.81 ± 1.03 ^ab^	5.10 ± 0.03 ^c^	5.83 ± 0.11 ^c^	5.21 ± 0.09 ^d^	4.87 ± 0.03 ^d^	8.50 ± 2.01 ^a^	6.00 ± 1.09 ^b^	7.20 ± 1.31 ^a^
	p-hydroxybenzoic acid	8.36 ± 0.68 ^a^	7.71 ± 0.81 ^b^	7.60 ± 0.31 ^b^	8.47 ± 0.96 ^a^	6.53 ± 2.02 ^bc^	8.02 ± 1.37 ^ab^	8.42 ± 1.28 ^a^	4.89 ± 0.02 ^d^	5.04 ± 0.06 ^c^
	vanillic acid	0.53 ± 0.05 ^c^	0.70 ± 0.07 ^b^	0.73 ± 0.04 ^b^	0.80 ± 0.02 ^a^	0.76 ± 0.13 ^b^	0.61 ± 0.02 ^bc^	0.34 ± 0.1 ^d^	0.64 ± 0.01 ^bc^	0.46 ± 0.06 ^d^
	chlorogenic acid	n.d.	0.16 ± 0.0 ^de^	0.60 ± 0.00 ^b^	0.62 ± 0.03 ^b^	0.32 ± 0.09 ^c^	0.58 ± 0.02 ^b^	0.15 ± 0.0 ^e^	1.02 ± 0.01 ^a^	0.27 ± 0.01 ^d^
	caffeic acid	0.88 ± 0.06 ^e^	0.80 ± 0.03 ^e^	2.03 ± 0.06 ^b^	2.38 ± 0.16 ^a^	2.11 ± 0.08 ^b^	1.85 ± 0.00 ^c^	0.80 ± 0.1 ^e^	1.71 ± 0.01 ^c^	1.38 ± 0.03 ^d^
	syringic acid	1.30 ± 0.04 ^c^	2.04 ± 0.01 ^a^	1.82 ± 0.03 ^a^	1.62 ± 0.02 ^ab^	1.50 ± 0.01 ^b^	1.63 ± 0.09 ^ab^	1.31 ± 0.5 ^c^	0.51 ± 0.03 ^d^	0.64 ± 0.09 ^d^
	p-coumaric acid	7.95 ± 0.36 ^a^	5.48 ± 0.18 ^bc^	6.27 ± 0.1 ^bc^	6.82 ± 0.63 ^b^	6.77 ± 0.46 ^b^	6.60 ± 0.38 ^b^	4.02 ± 0.87 ^d^	3.84 ± 0.97 ^d^	3.72 ± 0.86 ^d^
	ferulic acid	5.94 ± 0.87 ^a^	4.82 ± 0.86 ^a^	3.16 ± 0.36 ^bc^	3.20 ± 0.45 ^b^	3.24 ± 0.25 ^b^	3.07 ± 0.97 ^bc^	2.33 ± 0.63 ^d^	2.97 ± 0.17 ^c^	2.54 ± 0.06 ^cd^
	rosmarinic acid	n.d.	1.17 ± 0.23 ^c^	2.22 ± 0.75 ^b^	n.d.	n.d.	n.d.	n.d.	3.21 ± 0.17 ^a^	0.51 ± 0.08 ^d^
Flavonoids	quercitrin	1.91 ± 0.75 ^a^	0.97 ± 0.16 ^b^	1.20 ± 0.15 ^ab^	2.10 ± 0.36 ^a^	1.59 ± 0.75 ^ab^	1.93 ± 0.73 ^a^	0.50 ± 0.06 ^c^	n.d.	0.25 ± 0.11 ^c^
	quercetin	1.15 ± 0.03 ^cd^	1.25 ± 0.05 ^c^	1.79 ± 0.08 ^b^	3.57 ± 0.96 ^a^	2.97 ± 0.04 ^a^	3.04 ± 0.27 ^a^	1.01 ± 0.03 ^e^	1.89 ± 0.03 ^b^	1.24 ± 0.07 ^c^
	naringenin	1.538 ± 0.011 ^c^	0.75 ± 0.1 ^d^	1.09 ± 0.05 ^cd^	3.38 ± 0.09 ^a^	3.24 ± 0.03 ^a^	3.14 ± 0.09 ^a^	0.41 ± 0.9 ^d^	2.20 ± 0.04 ^b^	0.60 ± 0.03 ^d^
	kaempferol	0.33 ± 0.03 ^d^	0.31 ± 0.08 ^d^	0.80 ± 0.1 ^c^	3.19 ± 0.09 ^a^	1.68 ± 0.01 ^b^	2.58 ± 0.06 ^a^	0.42 ± 0.04 ^d^	0.91 ± 0.06 ^c^	0.35 ± 0.09 ^d^
	galangin	0.06 ± 0.00 ^c^	n.d.	0.08 ± 0.00 ^c^	0.17 ± 0.00 ^b^	n.d.	0.08 ± 0.00 ^c^	n.d.	0.26 ± 0.00 ^a^	0.10 ± 0.00 ^b^
Stilbenes	resveratrol	0.20 ± 0.02 ^de^	n.d.	1.03 ± 0.02 ^b^	1.69 ± 0.07 ^a^	1.50 ± 0.05 ^a^	1.20 ± 0.06 ^b^	0.17 ± 0.01 ^e^	0.82 ± 0.02 ^cd^	0.35 ± 0.05 ^d^

The values are presented as the mean of three replications ± standard deviations. Values in the same row marked with different superscript non-capital letters indicate statistically significant differences between the identified polyphenolic compounds (determined by post hoc Tukey HSD test, *p* < 0.05); n.d.—non-detected.

**Table 3:** Inhibition zones of FJ plant extracts against different microorganisms (mm).

Sample	Bacterial Strains						Yeast
	*S. aureus*	*B. cereus*	*E. faecalis*	*E. coli*	*P. aeruginosa*	*S. enteritidis*	*C. albicans*
Merișor roots	24.54 ± 0.35 ^b^	16.23 ± 0.24 ^b^	20.50 ± 0.34 ^a^	27.75 ± 0.61 ^a^	21.05 ± 0.21 ^b^	22.78 ± 0.57 ^b^	16.25 ± 0.50 ^a^
Merișor rhizomes	22.43 ± 0.39 ^b^	14.13 ± 0.39 ^cd^	17.36 ± 0.25 ^c^	25.03 ± 0.31 ^ab^	17.90 ± 0.72 ^cd^	18.35 ± 0.56 ^c^	14.51 ± 0.23 ^b^
Merișor stems	15.23 ± 0.25 ^ef^	12.77 ± 0.28 ^d^	16.42 ± 0.21 ^c^	20.82 ± 0.11 ^c^	15.86 ± 0.29 ^d^	14.59 ± 0.32 ^e^	12.06 ± 0.33 ^c^
Merișor leaves	14.58 ± 0.40 ^f^	10.43 ± 0.17 ^f^	13.56 ± 0.42 ^cd^	16.53 ± 0.35 ^d^	14.72 ± 0.37 ^de^	10.67 ± 0.22 ^g^	11.25 ± 0.25 ^c^
Valea Vinului roots	28.05 ± 0.22 ^a^	17.50 ± 0.34 ^ab^	21.25 ± 0.38 ^a^	26.08 ± 0.45 ^a^	23.05 ± 0.25 ^a^	22.24 ± 0.45 ^b^	17.52 ± 0.35 ^a^
Valea Vinului rhizomes	27.03 ± 0.13 ^a^	15.17 ± 0.29 ^c^	19.25 ± 0.22 ^b^	23.50 ± 0.55 ^b^	19.24 ± 0.24 ^c^	20.65 ± 0.72 ^bc^	12.78 ± 0.22 ^c^
Valea Vinului stems	18.21 ± 0.26 ^d^	15.32 ± 0.22 ^bc^	15.25 ± 0.52 ^c^	17.15 ± 0.37 ^d^	16.48 ± 0.35 ^cd^	14.78 ± 0.23 ^e^	14.35 ± 0.46 ^b^
Valea Vinului leaves	16.15 ± 0.63 ^e^	9.28 ± 0.13 ^g^	10.50 ± 0.24 ^f^	15.35 ± 0.13 ^de^	12.17 ± 0.33 ^f^	12.75 ± 0.38 ^f^	10.21 ± 0.24 ^cd^
Bocsig roots	25.32 ± 0.21 ^c^	18.54 ± 0.37 ^a^	19.56 ± 0.11 ^b^	24.50 ± 0.24 ^b^	20.45 ± 0.18 ^b^	24.56 ± 0.19 ^a^	15.67 ± 0.34 ^a^
Bocsig rhizomes	24.78 ± 0.52 ^a^	15.35 ± 0.42 ^c^	17.55 ± 0.25 ^d^	21.65 ± 0.17 ^c^	22.54 ± 0.52 ^b^	19.35 ± 0.38 ^c^	12.89 ± 0.33 ^c^
Bocsig stems	18.78 ± 0. 11 ^d^	10.56 ± 0.38 ^f^	13.56 ± 0.41 ^d^	14.91 ± 0.32 ^e^	17.13 ± 0.27 ^cd^	17.33 ± 0.32 ^cd^	9.54 ± 0.19 ^d^
Bocsig leaves	18.23 ± 0.23 ^d^	9.53 ± 0.25 ^fg^	14.73 ± 0.34 ^cd^	16.52 ± 0.15 ^d^	13.77 ± 0.22 ^e^	16.63 ± 0.31 ^d^	8.52 ± 0.25 ^e^

The values are presented as the mean of three replications ± standard deviations. Well diameter: 6 mm. Values in the same column marked with different lowercase letters indicate statistically significant differences in inhibitory activities against the evaluated microorganisms (determined by post hoc Tukey HSD test, *p* < 0.05).

**Table 4:** Inhibition zones of FJH against different microorganisms (mm).

Sample	Bacterial Strain						Yeast
	*S. aureus*	*B. cereus*	*E. faecalis*	*E. coli*	*P. aeruginosa*	*S. enteritidis*	*C. albicans*
FJH 1	14.67 ± 0.56 ^cd^	11.53 ± 0.34 ^b^	14.78 ± 0.25 ^b^	10.45 ± 0.18 ^d^	10.34 ± 0.21 ^d^	12.65 ± 0.43 ^c^	9.24 ± 0.11 ^b^
FJH 2	14.51 ± 0.27 ^cd^	8.34 ± 0.14 ^d^	12.65 ± 0.18 ^c^	10.56 ± 0.25 ^d^	12.45 ± 0.16 ^c^	11.32 ± 0.29 ^c^	7.24 ± 0.98 ^d^
FJH 3	13.63 ± 0.41 ^d^	7.32 ± 0.12 ^e^	11.54 ± 0.11 ^d^	9.48 ± 0.29 ^e^	8.55 ± 0.38 ^e^	8.76 ± 0.81 ^d^	7.34 ± 0.10 ^d^
FJH 4	17.35 ± 0.12 ^bc^	7.55 ± 0.95 ^e^	6.45 ± 0.78 ^e^	9.54 ± 0.12 ^e^	6.12 ± 0.32 ^f^	7.54 ± 0.51 ^e^	6.34 ± 0.75 ^e^
FJH 5	16.78 ± 0.32 ^c^	10.06 ± 0.26 ^c^	12.65 ± 0.11 ^c^	11.34 ± 0.23 ^c^	6.750 ± 0.47 ^f^	8.50 ± 0.24 ^d^	7.54 ± 0.56 ^d^
FJH 6	15.23 ± 0.11 ^c^	9.53 ± 0.75 ^c^	10.34 ± 0.35 ^d^	12.21 ± 0.68 ^c^	10.35 ± 0.55 ^d^	14.24 ± 0.15 ^b^	8.33 ± 0.13 ^c^
FJH 7	21.56 ± 0.43 ^a^	15.54 ± 0.29 ^a^	16.73 ± 0.41 ^a^	19.95 ± 0.52 ^a^	18.65 ± 0.13 ^a^	19.25 ± 0.27 ^a^	14.54 ± 0.12 ^a^
FJH 8	18.23 ± 0.21 ^b^	12.97 ± 0.32 ^b^	13.06 ± 0.37 ^c^	14.83 ± 0.14 ^b^	13.51 ± 0.26 ^b^	10.87 ± 0.35 ^cd^	8.94 ± 0.45 ^bc^
FJH 9	14.78 ± 0.75 ^cd^	8.63 ± 0.29 ^d^	10.78 ± 0.50 ^d^	8.26 ± 0.34 ^f^	10.23 ± 0.49 ^d^	9.23 ± 0.17 ^cd^	6.26 ± 0.50 ^e^

The values are presented as the mean of three replications ± standard deviations. Well diameter: 6 mm. Values in the same column marked with different lowercase letters indicate statistically significant differences in inhibitory activities against the evaluated microorganisms (determined by post hoc Tukey HSD test, *p* < 0.05).

## Data Availability

Data are available upon request to the authors.

## Supplementary Materials

The following supporting information can be downloaded at: https://www.mdpi.com/article/10.3390/plants13131883/s1, Supplementary Table S1: Chromatographic and spectral identification parameters of analyzed compounds. Concentration range of calibration curves and detection limit of each standard; Supplementary Table S2: Minimum inhibitory concentration (MIC) (µg/mL) of FJ plant extracts against different microorganisms; Supplementary Table S3: Minimum inhibitory concentration (MIC) (µg/mL) of FJH against different microorganisms; Supplementary Table S4: Percentages of biofilm eradication after treatment with various concentrations of FJ plant extracts (%); Supplementary Table S5: Percentages of biofilm eradication after treatment with various concentrations of FJH extracts, (%).
