# Supplementary material for: Preliminary Identification and Quantification of Individual Polyphenols in Fallopia japonica Plants and Honey and Their Influence on Antimicrobial and Antibiofilm Activities
Source: Plants (Basel). 2024 Jul 8;13(13):1883. doi: 10.3390/plants13131883 (PMC11244575; doi:10.3390/plants13131883)
Supplement: Supplementary file 1 [file plants-13-01883-s001.zip › plants-3072674-supplementary.pdf]

Supplementary Table S1. Chromatographic and spectral identification parameters of analyzed compounds. Concentration range of calibration curves and detection limit of each standard.

Nr.crt	Compound	Rt (min)	UV-VIS bands of standard (nm)	λmax (nm)	R (mg/L)	LOD (mg/L)
**Phenolic acids**						
1	Gallic acid	5.1	270	270	0.5 - 100	0.25
2	Protocatechuic acid	8.1	259,293	259	0.5 - 100	0.30
3	p-hydroxybenzoic acid	12.1	255	255	0.5 - 100	0.08
4	Vanillic acid	15.7	258, 290sh	258	0.05 - 100	0.04
5	Chlorogenic acid	17.3	245sh, 296sh, 323	323	0.1 - 100	0.05
6	Caffeic acid	18.4	239sh, 296s, 323	323	0.2 - 100	0.10
7	Syringic acid	19.1	274	274	0.1 - 100	0.05
8	p-Coumaric acid	24.5	293sh, 308	308	0.5 - 100	0.10
9	Ferulic acid	27.9	239sh, 296sh, 323	323	0.5 - 100	0.10
10	Rosmarinic acid	32.7	250sh, 290sh, 320	320	0.5 - 100	0.10
**Flavonoids**						
11	Catechina	15.3	278	278	0.5 - 100	0.10
12	Epicatechin	23.0	278	278	0.1 - 100	0.08
13	Rutina	30.6	255, 354	255	0.5 - 100	0.25
14	Isoquercitrin	30.8	255, 353	255	0.5 - 100	0.10
15	Naringin	31.8	282, 326	282	0.5 - 100	0.10
16	Quercitrin	32.2	258, 350	258	0.1 - 100	0.05
17	Quercetin	35.5	255, 295sh, 372	372	0.5 - 100	0.10
18	Naringenin	37.0	289, 326sh	289	0.1 - 100	0.05
19	Kaempherol	38.7	248sh, 266, 295sh, 318sh, 366	366	0.05 - 100	0.03
20	Apigenin	40.0	266, 290sh, 337	337	0.5 - 100	0.08
21	Galangin	46.0	237sh, 263, 288sh, 310, 358	263	0.05 - 100	0.03
**Phenolic aldehide**						
22	Vanillin	19.8	278, 308	278	0.5 - 100	0.08
**Stilbene**						
23	Resveratrol	33.5	306	306	0.1 - 100	0.05

Rt – retention time; R – Concentration range; LOD – Limit of detection

Supplementary Table S2. Minimum inhibitory concentration (MIC) (µg/ml) of FJ plant extracts against different microorganisms

**Sample**	**Bacterial strain**						**Yeast**
	S. aureus	***B. cereus***	***E. fecalis***	***E. coli***	***P. aeruginosa***	***S. enteritidis***	***C. albicans***
**Merișor roots**	0.19	0.39	0.39	0.19	0.39	0.39	0.78
**Merișor rhizomes**	1.56	3.12	0.78	0.78	1.56	0.78	3.12
**Merișor stems**	0.78	1.56	0.78	0.39	1.56	0.78	3.12
**Merișor leaves**	0.39	3.12	3.12	0.78	3.12	3.12	3.12
**Valea Vinului roots**	0.19	0.39	0.78	0.19	0.19	0.78	0.39
**Valea Vinului rhizomes**	0.78	1.56	1.56	1.56	1.56	1.56	3.12
**Valea Vinului stems**	0.39	3.12	3.12	0.39	0.39	0.78	0.78
**Valea Vinului leaves**	0.39	0.39	0.78	0.78	0.39	1.56	1.56
**Bocsig roots**	0.19	0.78	0.39	0.19	0.39	0.39	0.39
**Bocsig rhizomes**	0.78	3.12	3.12	1.56	0.39	3.12	1.56
**Bocsig stems**	1.56	1.56	1.56	0.39	1.56	0.78	3.12
**Bocsig leaves**	0.39	3.12	3.12	0.78	3.12	1.56	0.78

Supplementary Table S3. Minimum inhibitory concentration (MIC) (μg/ml) of FJH against different microorganisms

Sample	Bacterial strain						Yeast
	S. aureus	*B. cereus*	*E. fecalis*	*E. coli*	*P. aeruginosa*	*S. enteritidis*	*C. albicans*
**FJH 1 (MM)**	1.56	6.25	6.25	3.12	1.56	1.56	12.50
**FJH 2 (MM)**	3.12	6.25	25.00	6.25	3.12	3.12	6.25
**FJH 3 (MM)**	6.25	6.25	12.50	12.50	6.25	6.25	6.25
**FJH 4 (SM)**	6.25	12.50	12.50	6.25	12.50	12.50	25.00
**FJH 5 (SM)**	3.12	6.25	6.25	6.25	6.25	6.25	12.50
**FJH 6 (SM)**	3.12	12.50	12.50	6.25	3.12	6.25	12.50
**FJH 7 (AR)**	0.78	3.12	3.12	0.78	1.56	1.56	3.12
**FJH 8 (AR)**	1.56	6.25	6.25	1.56	1.56	3.12	6.25
**FJH 9 (AR)**	12.50	25.00	25.00	6.25	6.25	6.25	25.00

Supplementary Table S4. Percentages of biofilm eradication after treatment with various concentrations FJ plant extracts (%)

Sample	Concentration	Biofilm inhibiting activities, (%)				Yeast
		S. aureus	*E. fecalis*	*E. coli*	*P. aeruginosa*	*C. albicans*
**Roots MM**	MIC	42.83±0.58	41.65±0.37	45.41±0.54	38.23±0.56	42.49±0.66
	MIC x 2	67.53±0.72	65.31±0.45	69.21±0.68	60.07±0.91	66.88±0.82
	MIC x 4	92.47±0.37	90.08±0.17	91.98±0.45	88.37±0.41	90.19±0.49
**Rhizomes MM**	MIC	43.54±0.23	40.38±0.12	44.78±0.45	36.42±0.12	40.25±0.60
	MIC x 2	69.31±0.65	63.87±0.35	67.94±0.13	62.12±0.55	65.12±0.48
	MIC x 4	90.45±0.38	89.08±0.93	91.45±0.29	88.57±0.72	89.02±0.11
**Stems MM**	MIC	27.41±0.54	25.63±0.58	25.83±0.58	20.32±0.56	24.83±0.58
	MIC x 2	56.21±0.68	51.31±0.72	52.53±0.72	45.18±0.76	50.66±0.25
	MIC x 4	78.98±0.45	76.49±0.37	78.47±0.37	69.74±0.42	75.47±0.37
**Leaves MM**	MIC	30.75±0.50	21.19±0.22	27.76±0.56	20.33±0.56	25.78±0.34
	MIC x 2	56.74±0.74	44.88±0.56	54.94±0.74	44.17±0.76	52.21±0.68
	MIC x 4	82.93±0.52	72.19±0.49	80.33±0.82	68.24±0.42	77.98±0.45
**Roots SM**	MIC	46.76±0.50	40.03±0.55	34.75±0.12	35.29±0.59	40.83±0.58
	MIC x 2	70.94±0.74	65.12±0.42	60.88±0.55	62.17±0.76	61.53±0.72
	MIC x 4	93.23±0.52	92.01±0.65	88.29±0.46	85.14±0.69	89.47±0.37
**Rhizomes SM**	MIC	44.41±0.54	40.49±0.62	48.21±0.53	38.76±0.57	41.13±0.66
	MIC x 2	70.21±0.68	64.88±0.82	69.53±0.72	62.42±0.31	64.17±0.76
	MIC x 4	95.98±0.45	89.19±0.49	94.47±0.37	84.78±0.12	90.25±0.48
**Stems SM**	MIC	25.57±0.58	28.23±0.54	22.91±0.62	20.25±0.56	22.59±0.62
	MIC x 2	51.14±0.72	53.80±0.68	48.48±0.82	45.82±0.76	47.91±0.82
	MIC x 4	77.65±0.37	81.31±0.45	73.99±0.49	70.34±0.42	71.77±0.49
**Leaves SM**	MIC	30.89±0.50	24.38±0.58	26.17±0.54	22.43±0.66	27.91±0.50
	MIC x 2	56.46±0.74	50.03±0.72	52.19±0.68	43.28±0.54	54.05±0.74
	MIC x 4	84.97±0.52	74.52±0.37	77.36±0.45	69.84±0.42	80.21±0.52
**Roots AR**	MIC	47.41±0.54	37.23±0.56	45.55±0.70	43.78±0.61	40.23±0.56
	MIC x 2	72.21±0.68	65.55±0.75	70.08±0.24	64.78±0.99	63.17±0.82
	MIC x 4	94.98±0.45	86.24±0.42	93.21±0.15	91.55±0.57	88.24±0.42
**Rhizomes AR**	MIC	37.63±0.25	40.07±0.40	44.83±0.58	39.25±0.67	42.49±0.62
	MIC x 2	67.57±0.06	65.53±0.72	68.03±0.25	63.57±0.84	67.38±0.91
	MIC x 4	89.24±0.65	91.57±0.68	93.44±0.27	89.34±0.91	90.56±0.83
**Stems AR**	MIC	28.83±0.50	28.41±0.54	23.19±0.62	22.47±0.62	26.71±0.54
	MIC x 2	55.12±0.74	51.23±0.74	50.18±0.71	47.86±0.82	52.79±0.68
	MIC x 4	78.57±0.52	82.98±0.45	74.19±0.49	71.92±0.49	76.39±0.45
**Leaves AR**	MIC	29.75±0.50	27.40±0.53	28.14±0.54	20.73±0.56	20.91±0.68
	MIC x 2	59.94±0.74	55.68±0.70	54.02±0.68	45.28±0.76	48.09±0.82
	MIC x 4	81.23±0.52	87.23±0.52	79.78±0.45	70.65±0.42	73.17±0.49

Supplementary Table S5. Percentages of biofilm eradication after treatment with various concentrations of FJH extracts, (%)

Sample	Biofilm inhibiting activities, (%)					Yeast
	**Concentration**	***S. aureus***	***E. fecalis***	***E. coli***	***P. aeruginosa***	***C. albicans***
**FJH 1 (MM)**	MIC	34.65±0.19	27.65±0.47	30.64±0.78	26.45±0.25	25.50±0.13
	MIC x 2	60.14±0.79	51.67±0.21	59.32±0.46	55.71±0.74	50.36±0.11
	MIC x 4	87.23±0.15	81.54±0.31	88.54±0.50	82.77±0.84	83.45±0.78
**FJH 2 (MM)**	MIC	29.15±0.31	22.89±0.58	31.02±0.55	23.64±0.50	23.52±0.56
	MIC x 2	52.32±0.42	49.02±0.72	59.86±0.71	50.49±0.74	48.27±0.76
	MIC x 4	80.78±0.25	74.45±0.37	81.03±0.43	75.21±0.52	72.84±0.42
**FJH 3 (MM)**	MIC	34.21±0.63	39.21±0.33	32.78±0.42	24.97±0.59	20.43±0.56
	MIC x 2	61.05±0.92	57.89±0.28	59.92±0.74	50.62±0.79	45.27±0.76
	MIC x 4	86.47±0.35	83.54±0.51	84.01±0.31	75.71±0.47	70.84±0.42
**FJH 4 (SM)**	MIC	29.89±0.53	27.05±0.14	29.28±0.34	23.35±0.54	24.83±0.58
	MIC x 2	55.02±0.69	50.21±0.68	56.12±0.45	50.73±0.68	50.53±0.72
	MIC x 4	82.14±0.41	77.98±0.45	81.97±0.28	74.98±0.47	72.47±0.37
**FJH 5 (SM)**	MIC	33.75±0.18	25.39±0.97	29.41±0.42	23.67±0.91	24.71±0.32
	MIC x 2	60.56±0.82	50.74±0.46	62.68±0.15	52.45±0.79	55.49±0.50
	MIC x 4	86.73±0.56	80.45±0.19	86.98±0.68	81.67±0.34	79.82±0.56
**FJH 6 (SM)**	MIC	27.61±0.47	21.53±0.56	29.53±0.32	25.53±0.56	22.43±0.50
	MIC x 2	55.41±0.66	48.14±0.76	56.27±0.41	52.14±0.76	46.81±0.74
	MIC x 4	81.78±0.36	73.34±0.42	82.14±0.26	77.34±0.42	70.94±0.52
**FJH 7 (AR)**	MIC	35.52±0.46	29.76±0.37	37.25±0.82	32.89±0.65	30.78±0.24
	MIC x 2	61.34±0.25	59.61±0.83	65.34±0.79	60.35±0.29	60.34±0.93
	MIC x 4	89.45±0.56	85.12±0.75	90.75±0.45	86.25±0.34	85.67±0.82
**FJH 8 (AR)**	MIC	35.78±0.25	27.85±0.50	33.53±0.56	30.45±0.34	28.57±0.63
	MIC x 2	61.54±0.42	56.62±0.64	60.14±0.87	58.21±0.95	55.67±0.50
	MIC x 4	88.53±0.67	83.12±0.92	86.34±0.23	85.45±0.76	80.34±0.35
**FJH 9 (AR)**	MIC	30.15±0.58	26.19±0.62	29.82±0.29	25.49±0.30	23.19±0.56
	MIC x 2	56.73±0.72	51.88±0.82	56.53±0.37	51.92±0.39	47.58±0.75
	MIC x 4	84.47±0.45	77.19±0.49	82.39±0.23	79.02±0.27	74.19±0.49
